# Transcriptome-wide association study of coronary artery disease identifies novel susceptibility genes

**DOI:** 10.1007/s00395-022-00917-8

**Published:** 2022-02-17

**Authors:** Ling Li, Zhifen Chen, Moritz von Scheidt, Shuangyue Li, Andrea Steiner, Ulrich Güldener, Simon Koplev, Angela Ma, Ke Hao, Calvin Pan, Aldons J. Lusis, Shichao Pang, Thorsten Kessler, Raili Ermel, Katyayani Sukhavasi, Arno Ruusalepp, Julien Gagneur, Jeanette Erdmann, Jason C. Kovacic, Johan L. M. Björkegren, Heribert Schunkert

**Affiliations:** 1 Department of Cardiology, German Heart Center Munich, Technical University Munich, Lazarettstraße 36, 80636 Munich, Germany; 2 Fakultät für Informatik, Technische Universität München, Munich, Germany; 3 Deutsches Zentrum für Herz- und Kreislaufforschung (DZHK), Partner Site Munich Heart Alliance, Munich, Germany; 4 Department of Genetics and Genomic Sciences, Institute of Genomics and Multiscale Biology, Icahn School of Medicine at Mount Sinai, New York, NY 10029-6574 USA; 5 Department of Human Genetics, David Geffen School of Medicine, University of California, Los Angeles, CA USA; 6 Department of Medicine, David Geffen School of Medicine, University of California, Los Angeles, CA USA; 7 Department of Microbiology, Immunology and Molecular Genetics, David Geffen School of Medicine, University of California, Los Angeles, CA USA; 8 Department of Cardiac Surgery, The Heart Clinic, Tartu University Hospital, Tartu, Estonia; 9 Clinical Gene Networks AB, Stockholm, Sweden; 10 DZHK (German Research Centre for Cardiovascular Research), Partner Site Hamburg/Lübeck/Kiel, Lübeck, Germany; 11 Institute for Cardiogenetics, University of Lübeck, Lübeck, Germany; 12 Victor Chang Cardiac Research Institute, Darlinghurst, Australia; 13 St Vincent’s Clinical School, University of New South Wales, Sydney, Australia; 14 Icahn School of Medicine at Mount Sinai, Cardiovascular Research Institute, New York, NY 10029-6574 USA; 15 Department of Medicine, Huddinge, Karolinska Institutet, Karolinska Universitetssjukhuset, Stockholm, Sweden

**Keywords:** Coronary artery disease, Transcriptome-wide association study, Genome-wide association study, Genetically regulated expression

## Abstract

The majority of risk loci identified by genome-wide association studies (GWAS) are in non-coding regions, hampering their functional interpretation. Instead, transcriptome-wide association studies (TWAS) identify gene-trait associations, which can be used to prioritize candidate genes in disease-relevant tissue(s). Here, we aimed to systematically identify susceptibility genes for coronary artery disease (CAD) by TWAS. We trained prediction models of nine CAD-relevant tissues using EpiXcan based on two genetics-of-gene-expression panels, the Stockholm-Tartu Atherosclerosis Reverse Network Engineering Task (STARNET) and the Genotype-Tissue Expression (GTEx). Based on these prediction models, we imputed gene expression of respective tissues from individual-level genotype data on 37,997 CAD cases and 42,854 controls for the subsequent gene-trait association analysis. Transcriptome-wide significant association (i.e. *P* < 3.85e−6) was observed for 114 genes. Of these, 96 resided within previously identified GWAS risk loci and 18 were novel. Stepwise analyses were performed to study their plausibility, biological function, and pathogenicity in CAD, including analyses for colocalization, damaging mutations, pathway enrichment, phenome-wide associations with human data and expression-traits correlations using mouse data. Finally, CRISPR/Cas9-based gene knockdown of two newly identified TWAS genes, *RGS19* and *KPTN*, in a human hepatocyte cell line resulted in reduced secretion of APOB100 and lipids in the cell culture medium. Our CAD TWAS work (i) prioritized candidate causal genes at known GWAS loci, (ii) identified 18 novel genes to be associated with CAD, and iii) suggested potential tissues and pathways of action for these TWAS CAD genes.

**Supplementary Information:**

The online version contains supplementary material available at 10.1007/s00395-022-00917-8.

## Introduction

Coronary artery disease (CAD), a leading cause of premature death worldwide, is influenced by interactions of lifestyle, environmental, and genetic risk factors [43]. Genome-wide association studies (GWAS) have identified over 200 risk loci for CAD [11, 17, 35]. Most of them are located in non-coding regions which hampers their functional interpretation. Expression quantitative traits loci (eQTLs) to some extent explain the genomic effects of GWAS signals [19, 61, 64]. By leveraging effects of multiple *cis*-eQTL variants on gene expression, transcriptome-wide association studies (TWAS) search primarily for gene-trait associations. The approach first builds on prediction models of gene expression from reference panels that correlate genotype patterns with transcript levels in tissues which are relevant for the phenotype. Prediction models are then used to impute tissue-specific gene expression based on genotypes with a given trait in individuals of GWAS cohorts [21]. Since TWAS signals reflect association between transcriptome-wide genetically regulated expression (GReX) and traits or diseases, the approach can be used to prioritize candidate genes across disease-relevant tissues. Thereby, TWAS may point to causal genes at risk loci identified by GWAS and thus provide further insights into biological mechanisms [62, 70]. Moreover, TWAS increase the sensitivity to identify susceptibility genes missed by traditional GWAS analyses. Here we performed a TWAS to identify novel susceptibility genes for CAD comprising more than 80,000 individuals with genotype data along with validation and exploratory analyses for the associated genes.

## Materials and methods

### Prediction models of nine tissues based on two reference panels

The starting point of this investigation was two large human biobanks with individual-level data on genome-wide genotypes as well as mRNA expression levels in multiple tissues with relevance for CAD. These include atherosclerotic aortic wall (AOR), atherosclerotic-lesion-free internal mammary artery (MAM), liver (LIV), blood (BLD), subcutaneous fat (SF), visceral abdominal fat (VAF), and skeletal muscle (SKLM) in the Stockholm-Tartu Atherosclerosis Reverse Network Engineering Task (STARNET) [20], and AOR, LIV, BLD, SF, VAF, and SKLM in the Genotype-Tissue Expression (GTEx) [1] (Supplementary Table 1). Arterial wall coronary (COR) and tibial artery (TIB) datasets were only available in the GTEx. The pipeline used for training prediction models was EpiXcan which was built on the basis of PrediXcan but with improved prediction performance by integrating epigenomic annotation data into model-training process [21, 70]. The samples used for training models were restricted to European ancestry. We adopted the existing expression prediction models established by Zhang except COR and TIB tissues which were not covered yet [70].

We established predictive models for COR and TIB tissues using the same parameters as other tissues [70]. In brief, we first filtered the genotype and expression data of COR and TIB from GTEx v7 [1]. For genotype data, variants with call rate < 0.95, minor allele frequency (MAF) < 0.01, and Hardy Weinberg equilibrium (HWE) < 1e−6 were removed. For expression, we used quality-controlled data and performed sample-level quantile normalization and gene-level inverse quantile normalization using preprocess codes of PredicDB pipeline [21]. We then calculated SNP priors using hierarchical Bayesian model (qtlBHM) [40] that jointly analyzed epigenome annotations of aorta derived from Roadmap Epigenomics Mapping Consortium (REMC) [5], and eQTL statistics. The SNP priors (Supplementary Table 2), genotype data and expression data were jointly applied to tenfold cross-validated weighted elastic-net to train prediction models [70].

Both STARNET- and GTEx-based models were filtered by cross-validated prediction *R*^2^ > 0.01 [28, 68]. The summary statistics of sample sizes used for training models and the transcript numbers of genes covered by each predicting model are shown in Supplementary Table 1.

### GWAS cohorts

For the discovery cohort, individual-level genotyping data were collected from ten CAD GWAS, a subset of CARDIoGRAMplusC4D, including the German Myocardial Infarction Family Studies (GerMIFS) I–VII [16, 18, 38, 47, 48, 52, 56], Wellcome Trust Case Control Consortium (WTCCC) [7], LURIC [65], and Myocardial Infarction Genetics Consortium (MIGen) [2]. We used a part of individual-level data from UK Biobank (UKB) as the replication cohort [8], by extracting 20,310 CAD cases according to hospital episodes or death registries as reported and randomly selecting 25,000 non-CAD participants as controls. The detailed information about selection criteria of case and control were described at elsewhere [38]. In total, genotypes of 37,997 cases and 42,854 controls were included in our transcriptome-wide association studies (TWAS) of CAD (Supplementary Table 3). The preprocessing steps of genotyping data are as previously described [38].

### TWAS analysis

The transcriptome-wide association analysis was performed using prediction models of nine tissues for imputing individual-level GReX from CAD cases and controls of 11 GWAS cohorts and by association of these tissue-specific GReX with CAD risk in each cohort. To test the replicability of TWAS results, we used ten GWAS cohorts as discovery set and UKB as the replication set to test replicability within STARNET- and GTEx-based models, respectively. We compared the consistency of TWAS results between STARNET- and GTEx-based models of the six overlapping tissues using all genotype data. Then, we merged TWAS genes resulted from two reference-based panels as the final list. Finally, we annotated the TWAS genes list by over 200 CAD loci identified by GWASs [17, 35] using MAGMA [37]. Gene resided in the ± 1 Mb regions around known GWAS loci were marked as the known, otherwise genes were marked as the novel.

### Colocalization of the eQTL and GWAS signals

Colocalization analysis was performed using COLOC, a Bayesian statistical methodology that takes GWAS and eQTL data as inputs, and tests the posterior probabilities of hypothesis #4 (PP4) that there are shared casual variants for each locus [23]. The summary statistics of GWAS meta-analysis were obtained from CARDIoGRAMplusC4D Consortium [47], and the eQTL data of nine tissues from STARNET [20] and GTEx [1]. The significance threshold is PP4 > 0.55.

### Co-expression network for protein coding and lncRNA genes

We used RNA-seq data of STARNET [20] to calculate expression correlations between long non-coding RNA (lncRNA) genes and protein-coding genes in seven tissues. Co-expression pairs with absolute Pearson correlation coefficient larger than 0.4 were considered to be significant. The co-expression network was displayed by cytoscape [34].

### Gene set enrichment analyses

Pathway enrichment analysis was carried out using ClueGO (v2.5.2) [6], a plugin of cytoscape [34], based on collated gene sets from public databases including Gene Ontology (GO) [26], KEGG [30], Reactome [12], and WikiPathways [55]. Gene sets with false discovery rate (FDR) by right-sided hypergeometric test less than 0.05 were considered to be significant.

Furthermore, we also studied the diseases or traits associated with risk genes by performing disease enrichment analysis based on DisGeNET [50], the largest publicly available datasets of genes and variants association of human diseases. FDR < 0.05 was used for thresholding.

### Rare damaging variants association analysis

To investigate association of damaging variants in TWAS genes with CAD, we used whole-exome sequencing (WES) data of 200,632 participants from UKB [27]. The WES data were processed following the Functional Equivalence (FE) protocol. We performed quality control on the WES data by filtering variants with calling rate < 0.9 and variants with HWE < 1e−6. For the relevant traits, besides CAD, we considered i) three lifestyle factors including body mass index (BMI), diabetes, hypertension; ii) seven categories of blood lipids including low-density lipoproteins cholesterol (LDL-C), high density lipoproteins cholesterol (HDL-C), apolipoprotein A (APOA), apolipoprotein B (APOB), Lipoprotein(a) (LPA), total cholesterol (TC) and triglycerides (TG); iii) four inflammation related factors including C-reactive protein (CRP), lymphocyte count (Lymphocyte), monocyte count (Monocyte) and neutrophil count (Neutrophil). In total, 15 traits were studied.

We defined damaging variants as (i) MAF < 0.01; (ii) annotated into following one of the three classes: loss-of-function (LoF) variants (stop-gained, splice site disrupting, or frameshift variants), pathogenic variants in ClinVar [36], or missense variants predicted to be damaging by one of five computer prediction algorithms (LRT score, MutationTaster, PolyPhen-2 HumDiv, PolyPhen-2 HumVar, and SIFT). The Ensembl Variant Effect Predictor (VEP) [45] and its plugin loftee [31], and annotation databases dbNSFP 4.1a [14] and ClinVar (GRCh38) [36] were used for annotating damaging mutations.

For each analysis, samples were classified into carriers or noncarriers of the gene’s damaging mutations. For binary traits, we used Fisher’s exact test to check if there was incidence difference of mutation carrying between case and controls. For the quantitative traits, we used linear regression model with adjustments of sex, first five principal components, and lipid medication status to investigate the associations between mutation carrying status and traits. We used nominal significance threshold (*P* < 0.05), given that coding variants are rather rare, and the case–control sample sizes were not balanced which might increase false negative rate.

### Association of variants in novel genes with lipid traits

For 18 novel risk genes, we performed association analysis for variants located in respective loci (± 1 Mb) with lipid traits using genotyping data of UKB. The lipid traits include levels of LDL-C, HDL-C, APOA, APOB, LPA, TC, and TG. The variants were filtered by MAF > 0.01, and imputation info score > 0.4. The association test was performed using PLINK2 [10] with adjustment of sex, age, first five principal components, and lipid medication status. The numbers of independent SNPs were estimated using Genetic type 1 error calculator (GEC) tool [39].

### Expression-trait association study using mouse data

The hybrid mouse diversity panel (HMDP) is a set of 105 well-characterized inbred mouse strains on a 50% C57BL/6J genetic background [42]. To specifically study atherosclerosis in the HMDP, transgene implementation of human APOE-Leiden and cholesteryl ester transfer protein was performed, promoting distinct atherosclerotic lesion formation [4]. A Western diet containing 1% cholesterol was fed for 16 weeks. Subsequently, gene expression was quantified in aorta and liver of these mice and lesion size was assessed in the proximal aorta using oil red O staining. Fourteen other related traits were measured too, including liver fibrosed area, body weight, TC, VLDL-C (very low-density lipoprotein cholesterol) + LDL-C, HDL-C, TGs, unesterified cholesterol, free fatty acid (FFA), Il-1b, Il-6, Tnfa, Mcp-1, and M-csf. From HMDP, we extracted significant association pairs between TWAS genes and 15 risk traits by applying significance *P* < 0.05.

### Experimental validation of *KPTN* and *RGS19* in human cells

To knock down *KPTN* and *RGS19*, two sgRNAs targeting shared exons of all transcription isoforms were delivered by lentivirus into a Cas9-expression huh7, a human hepatoma cell line. Exon 4 of *KPTN* and exon 5 of *RGS19* were targeted by a dual CRISPR strategy to create a 40 bp and 130 bp frame shift deletion, respectively. SgRNAs were carried by Lenti-Guide-Puro vector (addgene, #52963) and infected cells were treated with 10 ug/ml puromycin treatment for 3 days to eliminate the negative cell. Positive targeted cells were expanded in culture and passaged for assays. Cells for measurement of secretive triglycerides, cholesterol, and APOB100 were cultured for 16 h in serum-free medium. Medium triglycerides and cholesterol were enriched for five times by vacuum centrifuge and measured with colorimetric kits, triglyceride (cobas), and CHOL2 (cobas), respectively. The amount of medium APOB100 was measured with an ELISA kit (MABTECH).

### RNA isolation and sequencing

Total RNA from huh7 cells was isolated using RNEasy Plus Mini Kit (Qiagen) (control cells, *n* = 3; knockout cells, *n* = 3). Quantity and quality of the isolated RNAs were measured by Fragment Analyzer (Agilent). RNA sequencing (RNA-seq) was performed by BGI TECH SOLUTIONS (HONGKONG) using strand specific library preparation with mRNA enrichment, paired-end sequencing with 100 bp read length on the DNBSEQ platform and 20 M clean read pairs per sample. Clean reads were mapped onto the GRCh38.p12. Expression quantifications, differential expression, and gene set enrichment were performed according to BGI RNA-seq pipeline.

## Results

### Transcriptome-wide significant genes for CAD

**Fig. 1:**
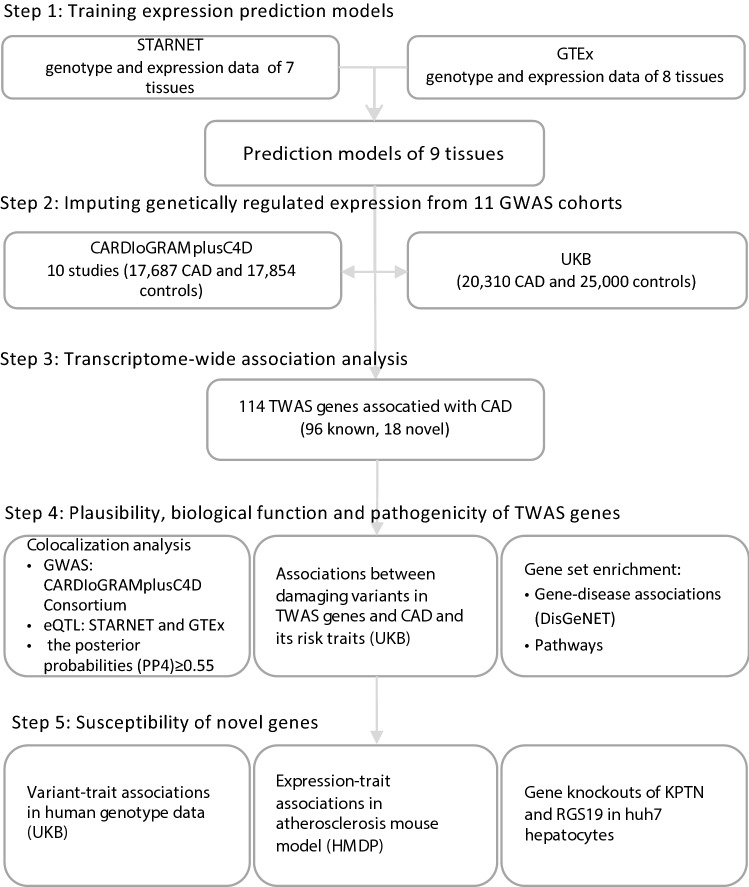
The study design. Step 1, we trained prediction models using EpiXcan from two eQTL panels, the Stockholm-Tartu Atherosclerosis Reverse Network Engineering Task (STARNET) and the Genotype-Tissue Expression (GTEx) for nine tissues. Step 2, the prediction models were applied to impute genetically regulated expression (GReX) from individual-level genotype data of ten CARDIoGRAMplusC4D sets and UK Biobank (UKB). Step 3, we associated transcriptome-wide GReX with risk of coronary artery disease (CAD) (Supplementary Results) and identified 114 transcriptome-wide significant genes (TWAS genes). Of these, 96 resided within genome-wide significant (GWAS) loci and 18 outside of known GWAS loci (novel genes). Step 4, we tested the plausibility of novel TWAS genes by conducting colocalization analysis and studying effects of damaging mutations, as well as gene set enrichment analyses. Step 5, we explored potential mechanisms of novel genes by testing association with risk traits of CAD in human genotype data of UKB, and association between expressions and risk traits of CAD in atherosclerosis mouse models from the Hybrid Mouse Diversity Panel (HMDP). Lastly, we carried out CRISPR/Cas9-based knockdown experiment for two novel genes *RGS19* and *KPTN* in human hepatocyte cell lines to experimentally validate related functions

The study design is shown in Fig. 1. Expression prediction models of nine tissues were derived from two reference panels, STARNET [20] and GTEx [1], using EpiXcan pipeline [70] (Materials and methods). We applied these models to impute transcriptome-wide GReX of nine tissues from individual-level genotype data of 11 GWAS cohorts (Supplementary materials; Supplementary Fig. 1–2; Supplementary Tables 1–3) [2, 7, 8, 16, 18, 38, 47, 52, 56, 65]. We next associated the GReX with CAD risk in each cohort (Supplementary materials). The results revealed replicability of TWAS genes when taking ten CARDIoGRAMplusC4D cohorts as discovery and UKB as replication set within the STARNET- and GTEx-based prediction models, respectively (Supplementary Fig. 2I–II; Supplementary Fig. 3). The results also showed consistency and complementarity of TWAS findings in six shared tissues between two reference-based prediction models (Supplementary Fig. 2III–IV; Supplementary Figs. 4–5). Therefore, we combined the results based on the two reference models for the final list of TWAS genes (Supplementary Fig. 2 V).

**Fig. 2:**
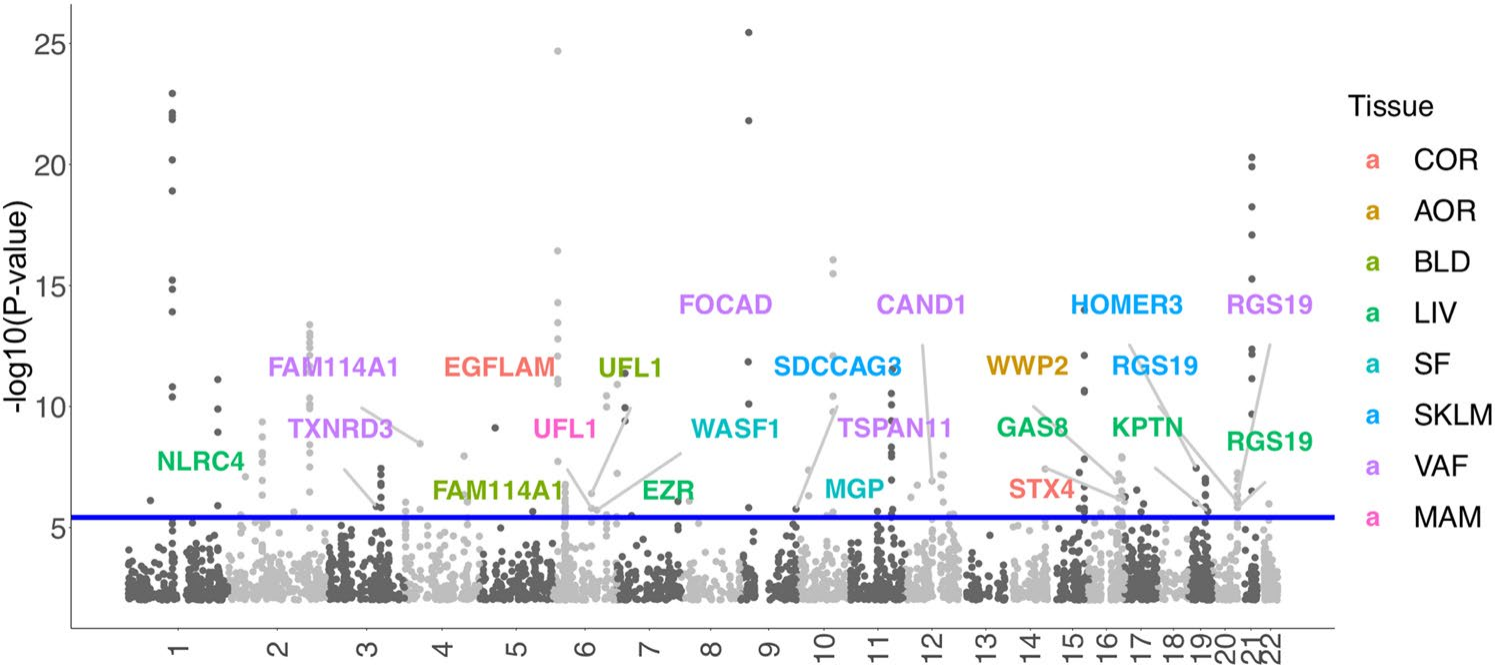
Manhattan plot of CAD TWAS results. The association results from STARNET- and GTEx-based models were integrated by lowest *P* values. The blue line marks *P* = 3.85e−6, i.e. transcriptome-wide significance. Each point corresponds to an association test between gene-tissue pair. 18 novel TWAS genes were highlighted. Supplementary Fig. 6 identifies all genes identified by their genetically-modulated association signals. The color code identifies the tissue in which the genes were differentially expressed by genetic means: *AOR* aorta, *COR* coronary artery, *MAM* mammary artery, *BLD* blood, *LIV* liver, *SF* subcutaneous fat, *VAF* visceral abdominal fat, *SKLM* skeletal muscle

From STARNET-based models 129 gene-tissues pairs and from GTEx-based models 106 gene-tissue pairs were significantly associated with CAD (Bonferroni-corrected significance based on 12,995 genes, *P* < 3.85e−6). Since 42 pairs overlapped between the two panels (Supplementary Fig. 5), the total number of gene-tissue pairs was 193. Given that some genes displayed association in multiple tissues, the final list of significant TWAS genes for CAD was 114 genes (Fig. 2; Supplementary Fig. 6; Supplementary Table 4). Of these, 95 gene-tissue association pairs were confirmed using another commonly used fine-mapping tool (COLOC) [23] with posterior probabilities of shared causal variants in each locus larger than 0.55 (PP4 > 0.55; Materials and methods; Supplementary Table 5; Supplementary Fig. 7).

**Fig. 3:**
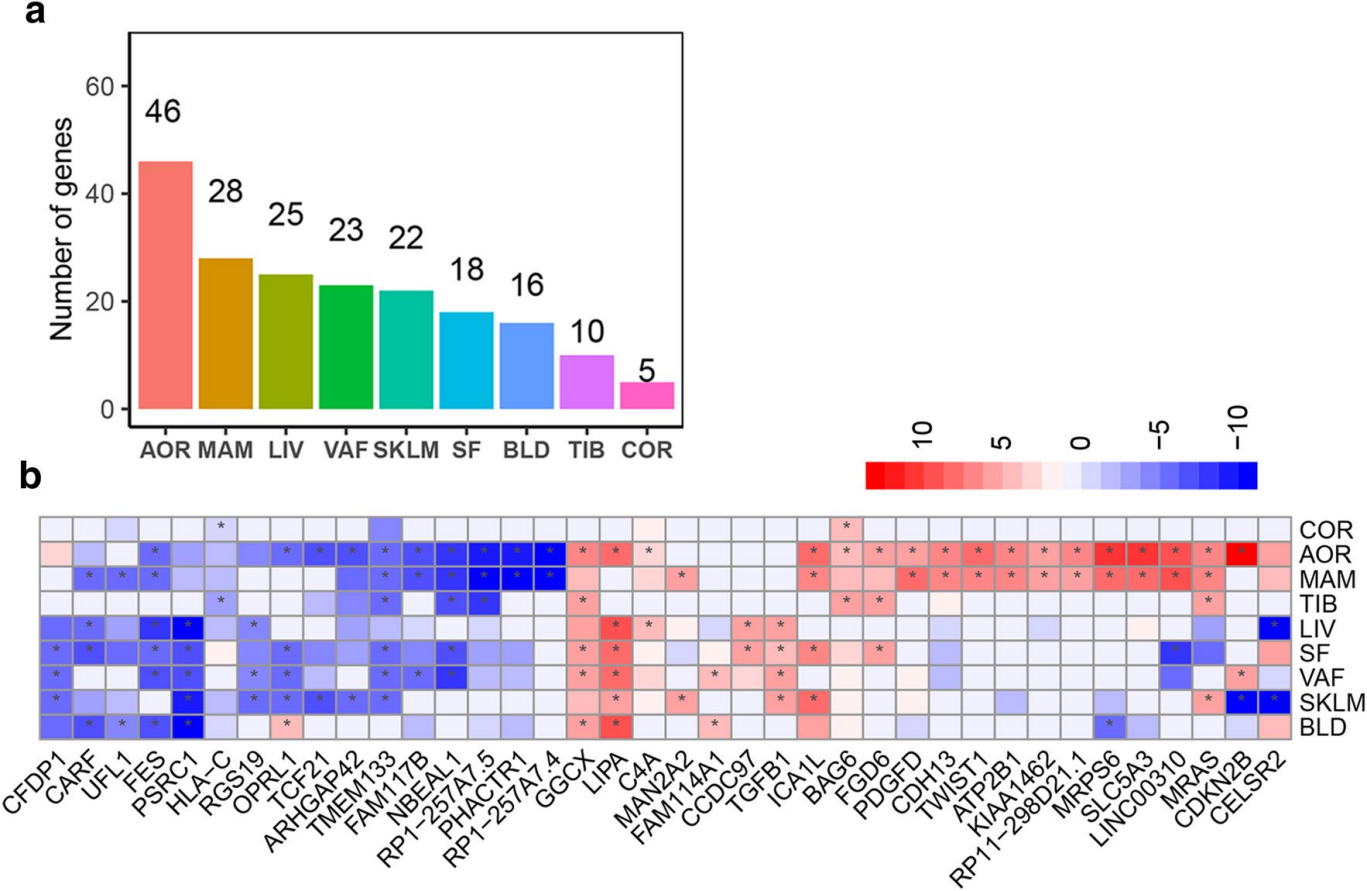
Tissue distribution of 114 TWAS genes of CAD. **a** Number of transcriptome-wide significant genes across tissues. **b** Heatmap plot of 38 genes identified in more than one tissues. The color codes indicate direction of effects. Cells marked with * represent significant gene-tissue pairs (*P* < 3.85e−6). *AOR* aorta, *COR* coronary artery, *MAM* mammary artery, *BLD* blood, *LIV* liver, *SF* subcutaneous fat, *VAF* visceral abdominal fat, *SKLM* skeletal muscle, *TIB* tibial artery

Of the 114 TWAS genes, 46 genes displayed genetically mediated differential expression in AOR, 28 in MAM, 25 in LIV, 23 in VAF, 22 in SKLM, 18 in SF, 16 in BLD, 10 in TIB, and 5 in COR (Fig. 3a). Most genes revealed significant associations in only a single tissue; 38 were significant in more than one, almost all having consistent directions of association between predicted expression levels and CAD across tissues (Fig. 3b).

Among the 114 genes, 102 were protein-coding and 12 were lnRNA genes (Supplementary Table 4). The STARNET data showed that most lncRNAs were positively co-expressed with a surrounding gene in affected tissues (Supplementary Fig. 8). *LINC00310* was the only exception, which displayed complex co-expression patterns with other genes.

Respective genes were found in 63 genomic regions, thus several regions represented multiple genes with significant associations. Six regions had multiple TWAS genes with shared GWAS and eQTL signals in respective tissues, like 1p13.3 and 2p33.2 (Supplementary Figs. 9–10; Supplementary Table 5). On the other hand, in 39 regions expression of only a single gene was found to be significantly associated, which makes these genes likely candidates for mediating causal effects, particularly, if these genes reside within GWAS risk loci for CAD (these genes are indicated in Supplementary Table 6).

Most TWAS genes (*n* = 96) could be positionally annotated to the 1 Mb region around one of the over 200 GWAS loci that are currently known to be genome-wide significantly associated with CAD [11, 17, 35]. Therefore, we marked these as known genes (Supplementary Table 6). On the other hand, 18 genes resided outside of these regions and were labeled as novel genes (Table 1). Most novel genes were tissue-specific, except *RGS19*, *FAM114A1* and *UFL1* which displayed evidence for differential expression in multiple tissues.

**Table 1:** 18 TWAS genes residing outside of published GWAS loci

Cytoband	Gene	Tissue	*Z* score	SE	*P* value	From^a^
2p22.3	*NLRC4*	LIV	− 3.383	0.044	3.04E−06	STARNET
3q21.3	*TXNRD3*	VAF	2.566	0.059	1.36E−06	STARNET
4p14	*FAM114A1*	VAF	4.026	0.050	3.44E−09	GTEx
4p14	*FAM114A1*	BLD	4.845	0.037	1.80E−06	GTEx
5p13.2	*EGFLAM*	COR	5.596	0.047	7.70E−10	GTEx
6q16.1	*UFL1*	MAM	− 5.246	0.038	1.62E−06	STARNET
6q16.1	*UFL1*	BLD	− 4.687	0.038	8.70E−05	STARNET
6q16.1	*UFL1*	BLD	− 4.955	0.042	3.96E−07	GTEx
6q21	*WASF1*	SF	4.320	0.059	1.91E−06	STARNET
6q25.3	*EZR*	LIV	− 3.187	0.025	3.53E−06	STARNET
9p21.3	*FOCAD*	VAF	8.348	0.068	1.44E−12	GTEx
9q34.3	*SDCCAG3*	SKLM	− 3.015	0.061	1.74E−06	STARNET
12p11.21	*TSPAN11*	VAF	2.285	0.065	1.79E−07	STARNET
12p12.3	*MGP*	SF	− 3.412	0.040	5.67E−07	GTEx
12q14.3	*CAND1*	VAF	− 2.355	0.030	1.19E−07	GTEx
16p11.2	*STX4*	COR	3.347	0.056	2.59E−06	GTEx
16q22.1	*WWP2*	AOR	4.491	0.029	5.67E−06	STARNET
16q22.1	*WWP2*	AOR	6.570	0.031	1.19E−07	GTEx
16q24.3	*GAS8*	LIV	0.189	0.041	8.32E−07	GTEx
19p13.11	*HOMER3*	SKLM	4.647	0.030	3.52E−08	GTEx
19q13.32	*KPTN*	LIV	− 3.076	0.076	2.17E−06	STARNET
20q13.33	*RGS19*	LIV	− 4.913	0.028	1.52E−06	GTEx
20q13.33	*RGS19*	VAF	− 4.545	0.030	4.63E− 07	GTEx
20q13.33	*RGS19*	SKLM	− 5.026	0.024	1.42E−06	STARNET
20q13.33	*RGS19*	SKLM	− 5.298	0.018	9.29E−07	GTEx

*TWAS* transcriptome-wide association study, *STARNET* the Stockholm-Tartu Atherosclerosis Reverse Network Engineering panel, *GTEx* the Genotype-Tissue Expression panel, *AOR* aorta, *COR* coronary artery, *MAM* mammary artery, *BLD* blood, *LIV* liver, *SF* subcutaneous fat, *VAF* visceral abdominal fat, *SKLM* skeletal muscle

^a^Association statistics from either STARNET- or GTEx-based models

### Pathways and diseases enriched by TWAS genes

We carried out two types of gene set enrichment tests to further study the biological relevance of genes giving signals in this TWAS. First, we studied disease-gene sets from the DisGeNET platform which is one of the largest publicly available collections of genes and variants associated with human diseases [50]. The results showed that genes discovered by TWAS were primarily enriched for CAD, coronary atherosclerosis, and hypercholesterolemia (Supplementary Table 7), adding to the plausibility of our TWAS findings.

In line with these results, gene set enrichment analysis based on GO [26], KEGG [30], Reactome [12], and WikiPathways [55] databases showed that the TWAS genes were highly enriched for pathways involved in cholesterol metabolism and regulation of lipoprotein levels. To a lesser extent, risk genes were enriched in regulation of blood pressure as well as development and morphogenesis of the heart and the aortic valve (Supplementary Table 8).

### Effects of damaging variants in TWAS genes

**Fig. 4:**
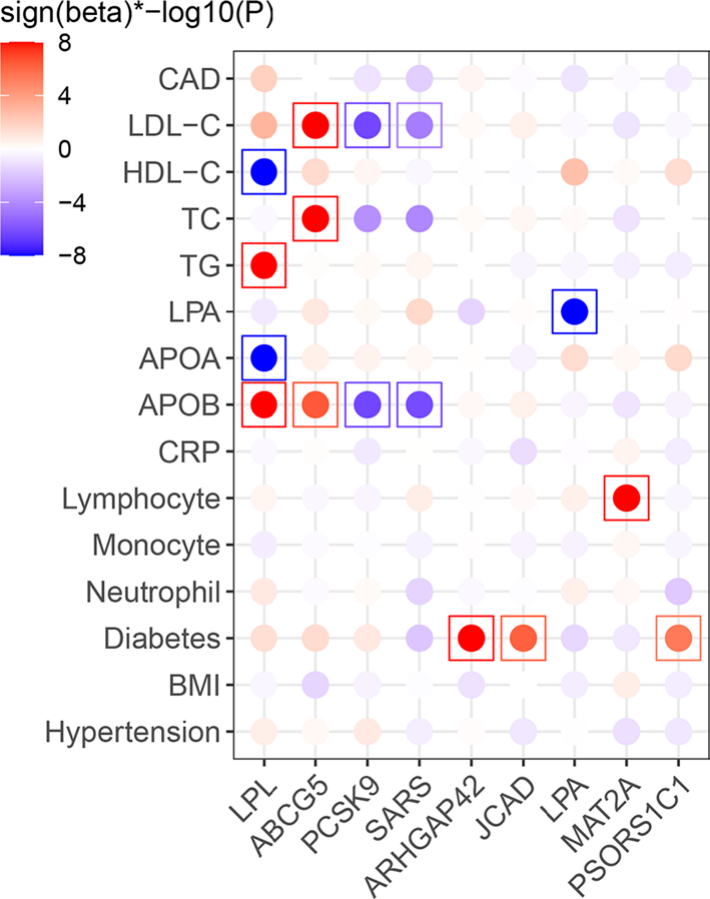
Effects of damaging variants in TWAS genes on CAD and its risk traits. Sign(beta)*−log10(p) displays direction and significance of gene-trait associations. When the Sign(beta)*−log10(*P*) > 8, they were trimmed to 8. The gene-trait association pairs reached Bonferroni-significance *P* < 3.44e−5 were highlighted in box. *CAD* coronary artery disease, *LDL-C* low-density lipoproteins cholesterol, *VLDL-C* very low-density lipoprotein cholesterol, *HDL-C* high density lipoproteins cholesterol, *APOA* apolipoprotein A, *APOB* apolipoprotein B, *TC* total cholesterol, *TG* triglycerides, *CRP* C-reactive protein, *BMI* body mass index

We next searched in exome-sequencing data of 200,643 participants from UKB for rare damaging variants in TWAS genes (either loss-of-function mutations or mutations predicted to be adverse by one of five in-silico methods, allele frequency < 0.01) (Materials and methods). In 97 genes we detected such variants. Expectedly these damaging mutations were very rare which limits the power of gene-based burden tests to observe association with risk of CAD or one of 14 CAD-related cardiometabolic traits we tested (15 traits in total). Nevertheless, associations of eight genes with risk traits reached Bonferroni-corrected significance (*P* < 3.44e−5; 0.05/97genes × 15traits) (Fig. 4; Supplementary Tables 9–10). Mutations of lipoprotein lipase (*LPL*), a critical regulator of lipid metabolism [29, 60], were evidently associated with lipid traits, including levels of HDL-C (beta = − 0.106; *P* = 4.54e−68), APOA (beta = − 0.062; *P* = 6.25e−47), APOB (beta = 0.025; *P* = 1.38e−12), and TG (beta = 0.241; *P* = 1.47e−68). *ABCG5*, encoding a sterol transfer protein [69], was associated with LDL-C (beta = 0.12; *P* = 3.66e−10), TC (beta = 0.16; *P* = 8.63e−10). *PCSK9*, a drug target for cholesterol lowering [13], was associated with LDL-C (beta = − 0.01; *P* = 4.29e−7) and APOB (beta = − 0.03; *P* = 4.4e−10). A mutation of *SARS* was associated with APOB (beta = − 0.02; *P* = 5.92e−7), *MAT2A* with lymphocyte counts (beta = 1.34; *P* = 3.41E−28), and *JCAD* (odds ratio [OR] = 1.31; 95% confidence interval [CI] 1.18–1.46; *P* = 5.77e−7) as well as *ARHGAP42* (OR = 2.08; 95% CI 1.65–2.59; *P* = 2.22e−9) were associated with risk of diabetes. We also observed nominally significant associations of several genes with CAD: *LPL* [29, 60] (OR = 1.168; CI 1.034–1.036; *P* = 0.016), *NOS3* [15] (OR = 1.143; 95% CI 1.109–1.279; *P* = 0.02), and *ADAMTS7* [32] (OR = 1.062; 95% CI 1.011–1.115; *P* = 0.016) (Supplementary Tables 9–10).

### Novel genes associated with risk factors in human and mouse data

**Fig. 5:**
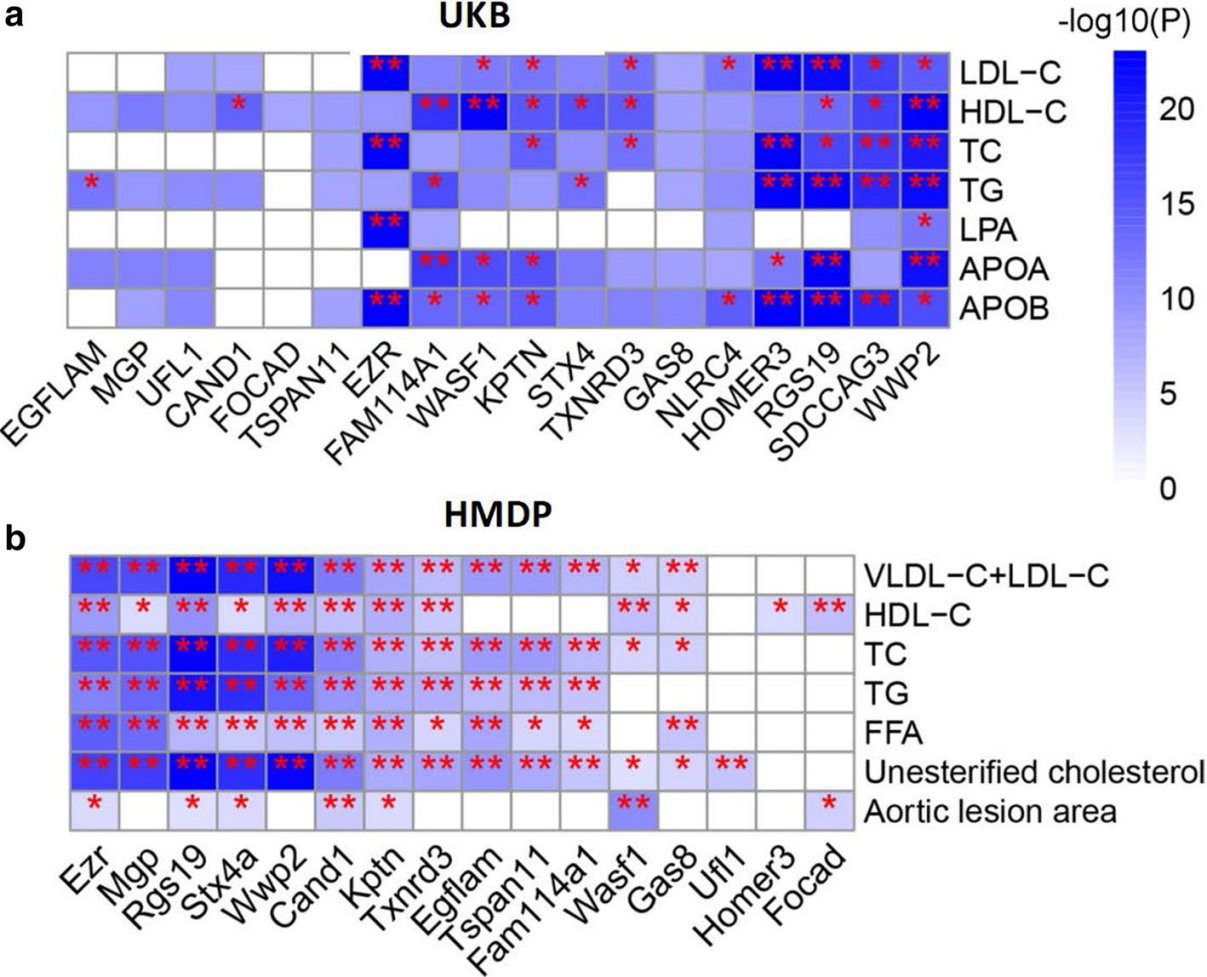
Novel risk genes were associated with lipid traits. **a** Data from UK Biobank (UKB) indicated that lead variants inside the boundary of risk genes were associated with lipid traits with Bonferroni-corrected significance levels (*P < 8.09e−6), or by genome-wide significance (**P < 5e−8). **b** Expression levels of novel genes were likewise associated with lipid traits and aortic lesion area in an atherosclerosis mouse model from the hybrid mouse diversity panel (HMDP). *P < 0.05; **FDR < 0.05. *LDL-C* low-density lipoproteins cholesterol, *VLDL-C* very low-density lipoprotein cholesterol, *HDL-C* high density lipoproteins cholesterol, *APOA* apolipoprotein A, *APOB* apolipoprotein B, *TC* total cholesterol, *TG* triglycerides, *FFA* free fatty acid

We next associated single nucleotide polymorphisms (SNPs) in the regions of ± 1 Mb around the 18 novel TWAS genes to study their associations with a series of lipid traits including LDL-C, HDL-C, APOA, APOB, LPA, TC, and TG in UKB (Materials and methods). There were 883 independent SNPs estimated by GEC. Bonferroni-corrected significance *P* < 8.09e−6 (0.05/883 × 7 lipid traits) was observed for numerous respective lead variants, of which *RGS19*, *SDCCAG3*, *EZR*, *HOMER3*, and *WWP2* reached genome-wide significant association (*P* < 5e−8) with multiple lipid traits (Fig. 5a; Supplementary Table 11).

Next, we extracted expression-trait association statistics of TWAS genes from HMDP, which brings together genotypes and expression data from atherosclerosis mouse models [42]. Based on the expression data from mouse aorta and liver tissues, 55 TWAS genes were significantly associated with aortic lesion area and 14 further cardiovascular traits (*P* < 0.05; Supplementary Table 12). Expression levels of seven novel genes, i.e. *Rgs19*, *Kptn*, *Ezr*, *Stx4a*, *Cand1*, *Focad*, and *Wasf1,* were associated with aortic lesion area (Fig. 5b), a commonly used measure for atherosclerotic plaque formation in mice. Additionally, we found the novel genes were associated with at least one lipid trait in the mouse model.

### Knockdown of *RGS19* and *KPTN* in human liver cells

Potential functional implications of all novel genes, based on the literature, are summarized in Supplementary Table 13. We additionally aimed to validate two exemplary novel TWAS genes by in vitro studies. Based on the above in-silico annotations we focused these studies on novel genes identified in liver with potential effects on lipids, the top risk factor for CAD (Fig. 5). Among the five genes identified in liver including *NLRC4*, *EZR*, *GAS8*, *KPTN*, and *RGS19*, the last two were, not only the least studied but also associated with nearly a full spectrum of lipid traits in human or mouse data (Fig. 5). In addition, both *KPTN* and *RGS19* are indeed expressed in hepatocyte (Supplementary Fig. 11a, b). Finally, both *KPTN* and *RGS19* are located within lipid loci identified recently in more than one million individuals [24]. Therefore, we decided to test the influence of *KPTN* and *RGS19* on lipid metabolism of liver cells.

**Fig. 6:**
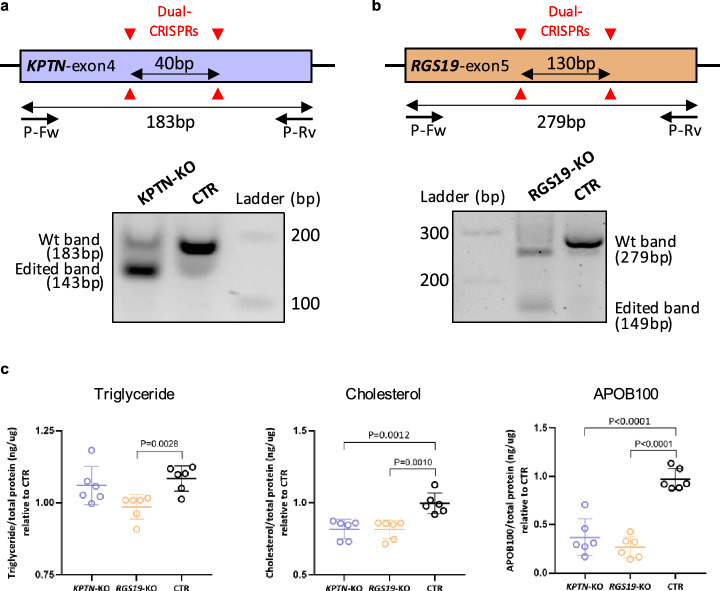
Targeting of *KPTN* and *RGS19* reduced lipids and APOB secretion of human liver cells. **a** Two sgRNAs were used to target the exon4 of *KPTN* (shared exon among isoforms) in a Cas9-expressing huh7 liver cell line. The dual CRISPR strategy created a 40 bp frame shift deletion in the gene and profound reduction of *KPTN* at both mRNA and protein levels (Supplementary Fig. 11c, d). The primers (P-Fw and P-Rv) used for analyzing the CRISPR editing as indicated. **b** The same strategy was used for *RGS19* targeting, which resulted in a 130 bp frame shift deletion in the gene, and reduction of mRNA and protein (Supplementary Fig. 11c, d). **c** Reduced triglyceride and cholesterol levels in knockout (KO) cell lines were detected by colorimetric method and APOB100 secretion was measured by human APOB100 Elisa (*n* = 6). Triglyceride, cholesterol, and APOB100 levels were normalized to total protein and compared between the KO and control (CTR) cell lines

We generated gene knockout (KO) huh7 cell lines by a dual CRISPR strategy (Materials and methods), which substantially reduced expression of the respective genes (Supplementary Fig. 11c, d). We measured secretion levels of TG, cholesterol and APOB in gene-targeted versus control cells. Notably, under normal circumstances, human hepatocytes synthesize cholesterol, assemble TG and APOB100, and secrete these particles in form of VLDL-C [58]. Compared to control huh7 cells, we found reduced APOB and cholesterol levels in culture medium of *KPTN*-KO cells (Fig. 6a, c). In culture medium of *RGS19*-KO cells we also detected reduced levels of APOB100, cholesterol, and TG (Fig. 6b, c), in line with strong associations of this gene with an array of lipid traits in both human genotyping and mouse expression data sets (Fig. 5).

We further corroborated our experimental results by performing RNA sequencing (RNA-seq) on *KPTN*-KO and *RGS19*-KO hepatocytes. In comparison to control cells, dysregulated genes in *KPTN*-KO and *RGS19*-KO hepatocytes (*P* < 0.05; Supplementary Tables 14–15) were indeed enriched for lipid metabolism (Supplementary Fig. 12). For *KPTN*-KO hepatocytes, the top four significantly enriched pathways plausibly contribute to CAD risk. Pathways ranked 1 and 3, ‘regulation of cholesterol esterification’ and ‘LDL particle remodeling’, strongly suggested that *KPTN* can affect CAD risk via cholesterol metabolism (Supplementary Fig. 12a, b). For *RGS19*-KO hepatocytes, the dysregulated genes were enriched for both cholesterol and triglycerides metabolisms (Supplementary Fig. 12c, d) and eight of the top ten significant enriched pathways were related to lipid metabolism, consistent with the reduced secretion of cholesterol and triglyceride of *RGS19*-KO cells (Fig. 6c).

## Discussion

In a stepwise approach, we first generated models which allow to predict gene expression based on genotypes in nine tissues. Next, we applied these models to individual-level genotype data on more than 80,000 CAD cases and controls to perform a transcriptome-wide association analysis. We identified 114 genes with differential expression by genetic means in CAD patients. Many signals were highly plausible as they resided within loci displaying genome-wide significant association with CAD by traditional GWAS. By in-silico analyses, these genes were markedly enriched in established pathways for the disease. Moreover, damaging variants in these genes showed association with CAD risk or its underlying traits in whole exome sequencing data from UKB. Importantly, we also identified 18 genes without prior evidence for their involvement in CAD by GWAS, many of which were found to be associated with lipid metabolism in human and mouse data.

Only a minority of genes residing within published CAD GWAS loci have been validated experimentally for their underlying causal role in atherosclerosis. Our data provide a substantial step towards prioritization of genes at respective GWAS loci [17, 35], because the TWAS association finding is based on expression levels of specific genes in defined tissues. In this respect, 46 genes identified by this TWAS are known for effects in pathophysiological pathways related to CAD, including lipid metabolism, inflammation, angiogenesis, transcriptional regulation, cell proliferation, NO signaling, and high blood pressure, to name a few (Supplementary Table 6), giving credibility to the association findings.

Interestingly, our TWAS uncovered eight novel gene-CAD associations in fat tissue, including *MGP* and *WASF1* in SF, and *CAND1*, *FAM114A1*, *FOCAD*, *RGS19*, *TSPAN11*, and *TXNRD3* in VAF, representing half of the novel genes. All these genes also showed significant association with multiple lipid traits in a mouse atherosclerosis model (Fig. 5b). Given many CAD patients that are overweight or obese, it will be of great interest to identify how these genes modify cardiometabolic traits leading to cardiovascular disorders. In this respect our TWAS could provide a list of candidate genes and related targetable cardiometabolic traits. In addition, it is of surprise to unveil 22 genes linking SKLM to CAD risk, and eight were unique to this tissue, including *HOMER3*, *SDCCAG3*, *MTAP*, *NME9*, *PSMA4*, *SLC2A12*, *UNC119B*, and *VAMP5*, the first two being novel. *SDCCAG3* or *ENTR1* encodes endosome associated trafficking regulator 1 and involves in recycling of *GLUT1* (glucose transporter type 1), supplying the major energy source for muscle contraction. SKLM-based metabolism may have a protective role in CAD as suggested by the many cardioprotective effects of sports [44, 54]. Gene targets enhancing SKLM function in this respect might be effective in CAD prevention, a field relatively unexplored thus far. Here, for the first time, quantitative traits regulated genes in SKLM were associated with CAD by TWAS, providing novel evidence for genes that could modulate CAD risk by their functions in SKLM.

Many novel TWAS genes revealed association with lipid traits in both genotype-trait data of human biobank and expression-trait data of atherosclerosis mouse model. For example, *KPTN* and *RGS19*, both novel genes displaying significant TWAS results for CAD—based on their genetically-modulated expression profiles of liver tissue—also showed significant association with various lipid traits as well as aortic lesion area in the atherosclerosis mouse model. Moreover, both gene loci harbor SNPs which are significantly associated with several lipids including LDL-C, HDL-C, TC, and/or TG in human genotype data. Based on these observations, we functionally validated the roles of these two novel genes by studying lipid levels in human liver cells, i.e. the tissue that displayed evidence for differential expression by TWAS. Indeed, we observed that knockout of the two genes lowered secretion of APOB and lipids into culture medium. *KPTN*, kaptin (actin binding protein), a member of the *KPTN*, *ITFG2*, *C12orf66*, and *SZT2* (KICSTOR) protein complex, is a lysosome-associated negative regulator of the mechanistic target of rapamycin complex 1 (mTORC1) signaling [67]. By investigating dysregulated genes of *KPTN*-KO hepatocytes, we found many genes of mTORC1 pathway to be upregulated (Supplementary Fig. 12b), including a subunit of mTORC1, namely, *MLST8* (MTOR associated protein, LST8 Homolog). Interestingly, many lysosome genes were also significantly upregulated including *LAMP1* (lysosomal associated membrane protein 1), *ACP2* (acid phosphatase 2, lysosomal), *AP1B1* (adaptor related protein complex 1 subunit beta 1), *ATP6V0C* (ATPase H+ transporting V0 subunit c), *CTNS* (cystinosin, lysosomal cystine transporter), *CTSA* (cathepsin A), and *CLTB* (clathrin light chain B). Lysosomes promote lipid catabolism and transport, and maintain cellular lipid homeostasis [57]. Activated mTORC1 located on lysosome membrane [67], acts as a sensor of lysosomal lipids [57], such as cholesterol and phosphatidic acid, which exert as building block for cellular and subcellular membrane system. In fact, cholesterol mediates mTORC1 activation at the lysosome [9]. The interaction of mTORC1 and lysosome may promote lipid-sensing and lipid-trafficking to support the function of other subcellular organelles [46, 57]. These results hint the enhanced cellular usage of cholesterol via mTOR-lysosome axis in *KPTN*-KO hepatocytes. In addition, several lipoprotein genes were downregulated as well, including *APOA1*, *APOA2*, *APOA4*, and *APOB*. Both processes might contribute to the reduced cholesterol secretion and the association with CAD.

*RGS19* belongs to the *RGS* (regulators of G-protein signaling) family, who are regulators for G-protein-coupled receptors (GPCRs) [49]. *RGS19* inhibits GPCR signal transduction by increasing the GTPase activity of G-protein alpha subunits, thereby transforming them into an inactive GDP-bound form [53, 59]. The targeting GPCR of *RGS19* has not been observed before, and how *RGS19* regulates lipid metabolism remains unclear. The *RGS19* locus was first reported to be associated with TC and TG in 2017 [33, 41]. We observed significant association of this gene with CAD and functionally validated its role in TG and cholesterol secretion. A potential mechanism could be related to PPARα pathway that regulates the expression of genes involving hepatic lipogenesis and lipid storage [63, 66]. PPARα also regulates cholesterol, bile acid homeostasis, and sphingolipid metabolism in the liver [22]. Many genes in PPARα pathway were significantly downregulated in *RGS19*-KO hepatocytes, including *FABP1* (also known as liver fatty acid binding protein), *PLTP* (phospholipid transfer protein), *APOA1*, *APOA2*, and *APOC3* (Supplementary Fig. 12d). *RGS19* is a regulator for G-protein-coupled receptors (GPCR). Interestingly, we found six dysregulated GPCRs in *RGS19*-KO hepatocytes, including, *ADGRL2*, *CELSR1*, *ADGRV1*, *OXER1*, *LGR5*, and *LGR4* (Supplementary Fig. 12d). Furthermore, one of them, *OXER1,* an activator PPARα [51], was also downregulated in *RGS19*-KO cells. All in all, one hypothesis could be that *RGS19* associated GPCR signaling affects the PPARα pathway, and thereby lipid metabolism and CAD risk. Previous and current studies concordantly suggest from different angles that *RGS19* has a role in lipid metabolism and our data further indicate that this function might meditate its effects on CAD risk.

There are certain limitations in our study. First, we observed that about 15% of gene-tissue pairs displayed some degree of heterogeneity in the association findings with CAD risk across the cohorts (Supplementary Table 4). While this number is relatively low and likely result from a play of chance when association findings are being compared across individuals with relatively small case–control samples, it might also indicate some degree of population specific effects within European ancestries from UK, Germany, France, and Italy. Second, since TWAS are strongly dependent on the reference panel linking genetic signatures with gene expression, it had to be expected that STARNET- and GTEx-based predictive models display some differences in gene-CAD associations. STARNET-based TWAS identified 129 gene-tissue pairs, whereas GTEx-based TWAS identified 106 gene-tissue pairs. Yet, 42 gene-tissue pairs were shared between the two analyses, and effect sizes for the shared genes were highly concordant (*ρ* = 0.97). An average of 62% overlapping genes was observed in the matched tissues of two reference-based models, and the resulting size of expression-CAD associations was linearly consistent with an average *ρ* = 0.72. The relatively small differences may be due to different sample sizes used for training predictive models [70], different disease states (subjects with and without CAD), intravital (STARNET) or post mortem (GTEx) sample collection, as well as different transcript abundance and genotype coverage leading to differences in expression associated SNPs in our reference panels [20, 25]. Given a fair consistency between the two data sources, we combined results derived from both panels to increase the power for capturing risk genes. Third, although TWAS facilitates candidate risk gene prioritization, co-regulation or co-expression *in cis* at a given locus limits the precise determination of the culprit gene [62]. Indeed, at 12 loci we observed signals for three or more TWAS genes. For instance, in LIV tissue TWAS identified five genes at 1p13.3, *ATXN7L2*, *CELSR2*, *PSMA5*, *PSRC1*, *SARS*, and *SORT1* which were co-regulated by same risk variant set, confusing prioritization of the causal gene. While *CELSR2*, *PSRC1*, and *SORT1* were previously shown to act on lipid metabolism [3], we found that damaging mutations in *SARS* were also associated with serum levels of HDL-C and APOA. Thus, a combined effects of some or all genes at this locus may contribute to the association signal. In addition, all lncRNA genes identified by our study displayed co-expression with their neighboring coding genes, which makes it difficult to determine their casual effects. Nevertheless, in combining TWAS data with other genetic analyses, e.g. effects of damaging mutations, genetic association with other phenotypes and expression-traits association statistics, we aimed to improve risk gene prioritization, and to provide deeper insights of possible disease-causing mechanisms. For instance, *LPL* is well-known for its protective role against CAD by lowering lipids [29, 60], and our analyses showed that damaging *LPL* mutations were associated with higher lipid levels. Last, as with all statistical methods, there are certain limitations and assumptions associated with TWAS. Further evolution and improvement of these methods, as well as functional validation experiments, will assuredly improve the accuracy of these studies.

In summary, our TWAS study based on two genetics-of-gene-expression panels identified 114 gene expression-CAD associations, of which 18 were novel. The extended analyses with multiple datasets supported the reliability of the CAD TWAS signals in prioritizing candidate risk genes and identifying novel associations in a tissue-specific manner. Functional validation of two novel genes, *RGS19* and *KPTN,* lend support to our TWAS findings and provide strong evidence for their role in lipid metabolism. Thus, our study created a set of gene-centered and tissue-annotated associations for CAD, providing insightful guidance for further biological investigation and therapeutic development.

## Supplementary Information

Below is the link to the electronic supplementary material.

Supplementary file1 (DOCX 60294 KB)

Supplementary file2 (XLSX 230 KB)

## Declarations

### Tools and data

EpiXcan pipeline: https://bitbucket.org/roussoslab/epixcan/src/master/, and predictive models based on STARNET and GTEx databases: http://predictdb.org/.

PrediXcan pipeline: https://github.com/hakyim/PrediXcan.

qtlBHM: https://github.com/rajanil/qtlBHM.

STARNET database: https://www.ncbi.nlm.nih.gov/projects/gap/cgi-bin/study.cgi?study_id=phs001203.v1.p1. Project ID: 13585.

GTEx database: https://www.ncbi.nlm.nih.gov/projects/gap/cgi-bin/study.cgi?study_id=phs000424.v8.p2. Project ID: 20848.

UK Biobank: https://www.ukbiobank.ac.uk/. Project ID: 25214.

MAGMA: https://ctg.cncr.nl/software/magma.

R package for colocalization analysis, coloc: https://cran.r-project.org/web/packages/coloc/vignettes/vignette.html.

DisGeNET: https://www.disgenet.org/.

CARDIoGRAMplusC4D Consortium: http://www.cardiogramplusc4d.org/.
