# Publisher Correction: Transcriptome-wide association study of coronary artery disease identifies novel susceptibility genes

**DOI:** 10.1007/s00395-022-00923-w

**Published:** 2022-04-05

**Authors:** Ling Li, Zhifen Chen, Moritz von Scheidt, Shuangyue Li, Andrea Steiner, Ulrich Güldener, Simon Koplev, Angela Ma, Ke Hao, Calvin Pan, Aldons J. Lusis, Shichao Pang, Thorsten Kessler, Raili Ermel, Katyayani Sukhavasi, Arno Ruusalepp, Julien Gagneur, Jeanette Erdmann, Jason C. Kovacic, Johan L. M. Björkegren, Heribert Schunkert

**Affiliations:** 1 Department of Cardiology, German Heart Center Munich, Technical University Munich, Lazarettstraße 36, 80636 Munich, Germany; 2 Fakultät für Informatik, Technische Universität München, Munich, Germany; 3 Deutsches Zentrum für Herz- und Kreislaufforschung (DZHK), Partner Site Munich Heart Alliance, Munich, Germany; 4 Department of Genetics and Genomic Sciences, Institute of Genomics and Multiscale Biology, Icahn School of Medicine at Mount Sinai, New York, NY 10029-6574 USA; 5 Department of Human Genetics, David Geffen School of Medicine, University of California, Los Angeles, CA USA; 6 Department of Medicine, David Geffen School of Medicine, University of California, Los Angeles, CA USA; 7 Department of Microbiology, Immunology and Molecular Genetics, David Geffen School of Medicine, University of California, Los Angeles, CA USA; 8 Department of Cardiac Surgery, The Heart Clinic, Tartu University Hospital, Tartu, Estonia; 9 Clinical Gene Networks AB, Stockholm, Sweden; 10 DZHK (German Research Centre for Cardiovascular Research), Partner Site Hamburg/Lübeck/Kiel, Lübeck, Germany; 11 Institute for Cardiogenetics, University of Lübeck, Lübeck, Germany; 12 Victor Chang Cardiac Research Institute, Darlinghurst, Australia; 13 St Vincent’s Clinical School, University of New South Wales, Sydney, Australia; 14 Icahn School of Medicine at Mount Sinai, Cardiovascular Research Institute, New York, NY 10029-6574 USA; 15 Department of Medicine, Huddinge, Karolinska Institutet, Karolinska Universitetssjukhuset, Stockholm, Sweden

## Publisher Correction: Basic Research in Cardiology (2022) 117:6 10.1007/s00395-022-00917-8

The original version of this article unfortunately contained a mistake. The presentation of Fig. 6 was incorrect. Please accept the inconvenience this may have caused.

The corrected Fig. 6 is given below.
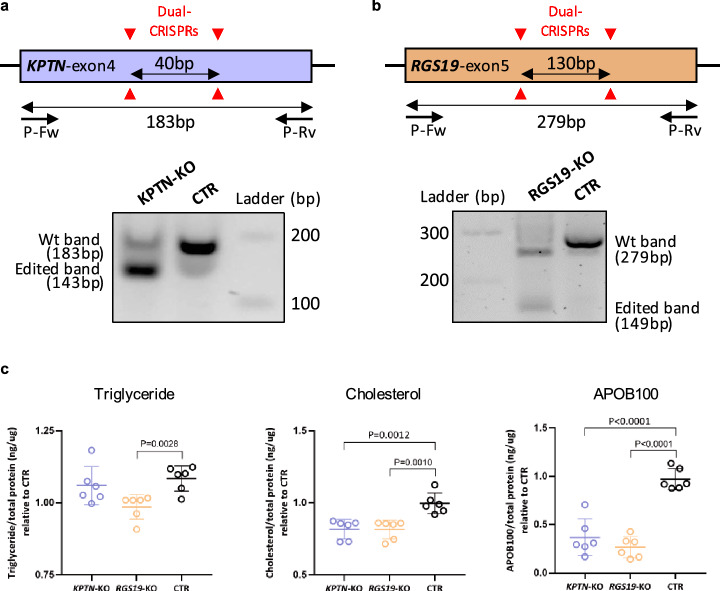


The original article has been corrected.

