# Supplementary material for: Transcriptome-wide association study of coronary artery disease identifies novel susceptibility genes
Source: Basic Res Cardiol. 2022 Feb 17;117(1):6. doi: 10.1007/s00395-022-00917-8 (PMC8852935; doi:10.1007/s00395-022-00917-8)
Supplement: Supplementary file 1 — Supplementary file1 (DOCX 60294 KB) [file 395_2022_917_MOESM1_ESM.docx]

Transcriptome-wide association study of coronary artery disease identifies novel susceptibility genes

Ling Li^1,2,3†^; Zhifen Chen^1,3†^; Moritz von Scheidt^1,3^; Shuangyue Li^1,3^; Andrea Steiner^1,3^; Ulrich Güldener^1,3^; Simon Koplev^4^; Angela Ma^4^; Ke Hao^4^; Calvin Pan^5^; Aldons J. Lusis^5,6,7^; Shichao Pang^1,3^; Thorsten Kessler^1,3,7^; Raili Ermel^8^; Katyayani Sukhavasi^8^; Arno Ruusalepp^8,9^; Julien Gagneur^2^; Jeanette Erdmann^10,11^; Jason C. Kovacic^12,13^; Johan L.M. Björkegren^4,9,14^; Heribert Schunkert^1,3^

^1^ Department of Cardiology, Deutsches Herzzentrum München, Technische Universität

München, Germany

^2^ Fakultät für Informatik, Technische Universität München, Germany

^3^ Deutsches Zentrum für Herz- und Kreislaufforschung (DZHK), partner site Munich Heart Alliance, Munich, Germany

^4^ Department of Genetics & Genomic Sciences, Institute of Genomics and Multiscale Biology, Icahn School of Medicine at Mount Sinai, New York, NY, 10029-6574, USA

^5^ Department of Human Genetics, David Geffen School of Medicine, University of California, Los Angeles, California, USA

^6^ Departments of Medicine, David Geffen School of Medicine, University of California, Los Angeles, California, USA

^7^ Departments of Microbiology, Immunology and Molecular Genetics, David Geffen School of Medicine, University of California, Los Angeles, California, USA

^8^ Department of Cardiac Surgery and The Heart Clinic, Tartu University Hospital, Tartu, Estonia

^9^ Clinical Gene Networks AB, Stockholm, Sweden

^10^ DZHK (German Research Centre for Cardiovascular Research), Partner Site Hamburg/Lübeck/Kiel, Lübeck, Germany.

^11^ Institute for Cardiogenetics, University of Lübeck, Lübeck, Germany.

^12^ Victor Chang Cardiac Research Institute, Darlinghurst, Australia; and St Vincent's Clinical School, University of New South Wales, Australia

^13^ Cardiovascular Research Institute, Icahn School of Medicine at Mount Sinai, New York, NY, 10029-6574, USA

^14^ Department of Medicine, Huddinge, Karolinska Institutet, Karolinska Universitetssjukhuset, Stockholm, Sweden

^†^ These two authors contributed equally to this work.

**Address for correspondence:**

Heribert Schunkert, MD

German Heart Center Munich, Technical University Munich

Lazarettstraße 36, 80636 Munich, Germany

Tel.: +49 89 1218 4073 [schunkert@dhm.mhn.de](mailto:schunkert@dhm.mhn.de)

# Supplementary materials

## Transcriptome-wide association analysis for CAD

We applied prediction models of nine tissues trained by the EpiXcan pipeline[16] from two reference panels, the Stockholm-Tartu Atherosclerosis Reverse Network Engineering Task panel (STARNET) and the Genotype-Tissue Expression panel(GTEx)[6, 7]. STARNET is a genetics-of-gene-expression study on approximately 600 CAD patients undergoing open-heart surgery, during which seven tissues were collected: AOR, MAM, BLD, LIV, SF, VAF and SKLM[6]. GTEx is a comprehensive resource for genetics-of-gene-expression across 54 non-diseased tissue sites obtained post-mortem from nearly 1000 individuals[7]. In GTEx we studied six of the above tissues as well as the COR and TIB tissues, whereas MAM was not available (Materials and methods; Supplementary Tables 1-2). Together, we obtained predictive models from nine CAD-relevant tissues. Genes with cross-validated prediction R2>0.01 were kept (Supplementary Fig. 1)[8, 15]. STARNET-based models allowed to impute 12,995 unique gene expression signatures in seven tissues, and GTEx 12,964 unique gene expression signatures in eight tissues (Supplementary Table 1). Because of similar genes number covered by two reference-based models, the threshold of transcriptome-wide significant was set as Bonferroni-corrected significance *P*<3.85e-6 (0.05/12,995 or 0.05/12,964).

The transcriptome-wide association analyses were carried according to Supplementary Fig. 2. The prediction models of nine tissues from two reference panels were applied to impute genetically regulated expression (GReX) from genotype data of ten CARDIoGRAMplusC4D studies covering 17,687 CAD patients and 17,854 controls[1, 2, 4, 5, 9–14], and genotype data of UKB covering 20,310 CAD patients and 25,000 controls (Supplementary Table 3)[3]. Then the imputed GReX were used to test associations with CAD for each cohort.

Based on the association statistics, we first tested replicability of TWAS results within the STARNET- and GTEx-based prediction models using genotype data from ten CARDIoGRAMplusC4D as discovery set, and UKB as replication set (Supplementary Fig. 2 I-II). From STARNET-based models, we identified 49 genes representing 66 gene-tissue pairs reaching Bonferroni-corrected significance *P<*3.85e-6 in discovery set. Of these, 12 genes representing 18 gene-tissue pairs were replicated in UKB (*P<*3.85e-6), which was significantly more than expected by chance (binomial test *P=*3.96e-6), and 50 of 66 gene-tissue association pairs had directionally consistent effects (binomial test *P=*3.33e-5). We also found strong correlation of the effect sizes (*ρ*=0.74; *P=*1.3e-12; Supplementary Fig. 3a) between discovery and replication sets indicating good overall reproducibility of the STARNET-based models. From the GTEx-based models, 35 genes representing 47 gene-tissue pairs reached Bonferroni-corrected significance *P<*3.85e-6 in the discovery set, whereof 14 genes were significant also in replication set (binominal test *P=*5.20e-10). Like the STARNET-based models, 39 of 44 significant gene-tissue association pairs had consistent direction of effects with a Pearson´s coefficient of 0.75 between discover and replication sets (*P=*1.2e-9; Supplementary Fig. 3b).

To enlarge the statistical power, we then performed the meta-analysis on eleven cohorts for each tissue within panels (Supplementary Fig. 2III). Next, we tested the consistency between STARNET-TWAS and GTEx-TWAS statistics in six shared tissues of the two-reference panels (Supplementary Fig. 2 IV). We observed an average of 62% overlapping genes (Supplementary Table 1) and significant correlations in effect sizes in the six shared tissues (average Pearson’s coefficient ρ=0.72; *P<*1e-10; Supplementary Fig. 4). In the STARNET-based models, we identified 82 genes representing 129 gene-tissue pairs across seven tissues (*P<*3.85e-6). In the GTEx-based models, we identified 66 genes representing 106 gene-tissue pairs across eight tissues (*P<*3.85e-6). A total of 42 gene-tissue pairs were significant in both the STARNET- and GTEx-based models (Supplementary Fig. 5a). The overlapping genes were linearly consistent in both effect size (Pearson’s coefficient ρ=0.99; *P<*2.2e-16) and -log_10_P (Pearson´s coefficient ρ=0.82; *P<*4e-11) (Supplementary Fig. 5b). Overall, these results suggest, on the one hand, consistency between the two independent panels and, on the other hand, evidence for capturing complementary expression quantitative signals.

Therefore, we combined two lists as final TWAS genes list of CAD covering 114 genes representing 193 gene-tissue pairs (129 pairs from STARNET + 106 pairs from GTEx - 42 pairs overlapped) (Fig. 2; Supplementary Fig. 2 V; Supplementary Fig. 6; Supplementary Table 4).

## Heterogeneity of TWAS genes

We calculated heterogeneity of TWAS genes across the cohorts. We combined results of the German Myocardial Infarction Family Studies (GerMIFS) I-VII[1, 2, 4, 5, 9–14], Wellcome Trust Case Control Consortium (WTCCC)[2], LURIC[14], Myocardial Infarction Genetics Consortium (MIGen)[1], and UKB[3]. We observed heterogeneous effects in 15% gene-tissues pairs across the cohorts (*P*<0.01; Supplementary Table 4). The result also showed overall low heterogeneity for same gene-tissue pairs between STARNET- and GTEx-based models.

# Reference

1. Anderson CD, Rosand J, Chen EY, Tan CM, Kou Y, Duan Q, Wang Z, Meirelles G V, Clark NR, Ma’ayan A, Kim JY, Kim JY, Song KS, Lee YH, Seo JS, Jelinek J, Goldschmidt-Clermont PJ, Issa JP, Kim M, Long TI, Arakawa K, Wang R, Yu MC, Laird PW, Krizsan-Agbas D, Pedchenko T, Smith PG, Li C, Bazzano LA, Rao DC, Hixson JE, He J, Gu D, Gu CC, Shimmin LC, Jaquish CE, Schwander K, Liu DP, Huang J, Lu F, Cao J, Chong S, Lu X, Kelly TN, Sharma P, Kumar J, Garg G, Kumar A, Patowary A, Karthikeyan G, Ramakrishnan L, Brahmachari V, Sengupta S, Pedrinelli R, Ballo P, Fiorentini C, Denti S, Galderisi M, Ganau A, Germanò G, Innelli P, Paini A, Perlini S, Salvetti M, Zacà V, Besingi W, Johansson Å, Cao TH, Quinn PA, Sandhu JK, Voors AA, Lang CC, Parry HM, Mohan M, Jones DJL, Ng LL, Heart N, Deloukas P, Kanoni S, Willenborg C, Farrall M, Assimes TL, Thompson JR, Ingelsson E, Saleheen D, Erdmann J, Goldstein BA, Stirrups KK, König IR, Cazier J-B, Johansson Å, Hall AS, Lee J-YJ-Y, Willer CJ, Chambers JC, Esko T, Folkersen L, Goel A, Grundberg E, Havulinna AS, Ho WK, Hopewell JC, Eriksson N, Kleber ME, Kristiansson K, Lundmark P, Lyytikäinen L-P, Rafelt S, Shungin D, Strawbridge RJ, Thorleifsson G, Tikkanen E, Van Zuydam N, Voight BF, Waite LL, Zhang W, Ziegler A, Absher D, Altshuler D, Balmforth AJ, Barroso I, Braund PS, Burgdorf C, Claudi-Boehm S, Cox D, Dimitriou M, Do R, Doney ASF, Mokhtari NEE El, Eriksson P, Fischer K, Fontanillas P, Franco-Cereceda A, Gigante B, Groop L, Gustafsson S, Hager J, Hallmans G, Han B-G, Hunt SE, Kang HM, Illig T, Kessler T, Knowles JW, Kolovou G, Kuusisto J, Langenberg C, Langford C, Leander K, Lokki M-L, Lundmark A, McCarthy MI, Meisinger C, Melander O, Mihailov E, Maouche S, Morris AD, Müller-Nurasyid M, Nikus K, Peden JF, Rayner NW, Rasheed A, Rosinger S, Rubin D, Rumpf MP, Schäfer A, Sivananthan M, Song C, Stewart AFR, Tan S-T, Thorgeirsson G, Schoot CE van der, Wagner PJ, Wells G a., Wild PS, Yang T-P, Amouyel P, Arveiler D, Basart H, Boehnke M, Boerwinkle E, Brambilla P, Cambien F, Cupples AL, de Faire U, Dehghan A, Diemert P, Epstein SE, Evans A, Ferrario MM, Ferrières J, Gauguier D, Go AS, Goodall AH, Gudnason VV, Hazen SL, Holm H, Iribarren C, Jang Y, Kähönen M, Kee F, Kim H-S, Klopp N, Koenig W, Kratzer W, Kuulasmaa K, Laakso M, Laaksonen R, Lee J-YJ-Y, Lind L, Ouwehand WH, Parish S, Park JE, Pedersen NL, Peters A, Quertermous T, Rader DJ, Salomaa V, Schadt E, Shah SH, Sinisalo J, Stark K, Stefansson K, Trégouët D-A, Virtamo J, Wallentin L, Wareham NJN, Zimmermann ME, Nieminen MS, Hengstenberg C, Sandhu MS, Pastinen TM, Syvänen A-C, Hovingh GK, Dedoussis G, Franks PW, Lehtimäki T, Metspalu A, Zalloua PA, Siegbahn A, Schreiber S, Ripatti S, Blankenberg SS, Perola M, Clarke R, Boehm BO, O’Donnell CJ, Reilly MP, März W, Collins R, Kathiresan S, Hamsten A, Kooner JS, Thorsteinsdottir U, Danesh J, Palmer CNA, Roberts R, Watkins H, Schunkert H, Samani NJ, Willer CJ, Schmidt EM, Al E, Facp AFR, Rodriguez-lopez L, Vargas-ayala G, Mc SH, Serna DC, Lozano-nuevo JJ, Rubio-guerra AF, Studies TIC for BPG-WA, Franks WT, Zhou DH, Wylie BJ, Money BG, Graesser DT, Frericks HL, Sahota G, Rienstra CM, Rakyan VK, Down TA, Balding DJ, Beck S, Hager J, Kamatani Y, Cazier J-B, Youhanna S, Ghassibe-Sabbagh M, Platt DE, Abchee AB, Romanos J, Khazen G, Othman R, Badro DA, Haber M, Salloum AK, Douaihy B, Shasha N, Kabbani S, Sbeite H, Chammas E, Bayeh H el, Rousseau F, Zelenika D, Gut I, Lathrop M, Farrall M, Gauguier D, Zalloua PA, Myocardial Infarction Genetics C, Kathiresan S, Voight BF, Purcell S, Musunuru K, Ardissino D, Mannucci PM, Anand SS, Engert JC, Samani NJ, Schunkert H, Erdmann J, Reilly MP, Rader DJ, Morgan T, Spertus JA, Stoll M, Girelli D, McKeown PP, Patterson CC, Siscovick DS, O’Donnell CJ, Elosua R, Peltonen L, Salomaa V, Schwartz SM, Melander O, Altshuler D, Merlini PA, Berzuini C, Bernardinelli L, Peyvandi F, Tubaro M, Celli P, Ferrario MM, Fetiveau R, Marziliano N, Casari G, Galli M, Ribichini F, Rossi M, Bernardi F, Zonzin P, Piazza A, Yee J, Friedlander Y, Marrugat J, Lucas G, Subirana I, Sala J, Ramos R, Meigs JB, Williams G, Nathan DM, MacRae CA, Havulinna AS, Berglund G, Hirschhorn JN, Asselta R, Duga S, Spreafico M, Daly MJ, Nemesh J, Korn JM, McCarroll SA, Surti A, Guiducci C, Gianniny L, Mirel D, Parkin M, Burtt N, Gabriel SB, Thompson JR, Braund PS, Wright BJ, Balmforth AJ, Ball SG, Hall AS, Wellcome Trust Case Control C, Linsel-Nitschke P, Lieb W, Ziegler A, Konig IR, Hengstenberg C, Fischer M, Stark K, Grosshennig A, Preuss M, Wichmann H-EE, Schreiber S, Ouwehand WH, Deloukas P, Scholz M, Cambien F, Li M, Chen Z, Wilensky R, Matthai W, Qasim A, Hakonarson HH, Devaney JM, Burnett MS, Pichard AD, Kent KM, Satler L, Lindsay JM, Waksman R, Knouff CW, Waterworth DM, Walker MC, Mooser V, Epstein SE, Scheffold T, Berger K, Huge A, Martinelli N, Olivieri O, Corrocher R, McKeown PP, Erdmann E, Konig IR, Holm H, Thorleifsson G, Thorsteinsdottir U, Stefansson K, Do R, Xie C, Siscovick DS, Liang L, Willis-Owen SAG, Laprise C, Wong KCC, Davies GA, Hudson TJ, Binia A, Hopkin JM, Yang I V., Grundberg E, Busche S, Hudson M, Rönnblom L, Pastinen TM, Schwartz DA, Lathrop GM, Moffatt MF, Cookson WOCM, Manolio TA, Collins FS, Cox NJ, Goldstein DB, Hindorff LA, Hunter DJ, McCarthy MI, Ramos EM, Cardon LR, Chakravarti A, Cho JH, Guttmacher AE, Kong A, Kruglyak L, Mardis E, Rotimi CN, Slatkin M, Valle D, Whittemore AS, Boehnke M, Clark AG, Eichler EE, Gibson G, Haines JL, Mackay TF, McCarroll SA, Visscher PM, Mayer B, Erdmann J, Schunkert H, Myocardial Infarction Genetics C, Kathiresan S, Voight BF, Purcell S, Musunuru K, Ardissino D, Mannucci PM, Anand SS, Engert JC, Samani NJ, Schunkert H, Erdmann J, Reilly MP, Rader DJ, Morgan T, Spertus JA, Stoll M, Girelli D, McKeown PP, Patterson CC, Siscovick DS, O’Donnell CJ, Elosua R, Peltonen L, Salomaa V, Schwartz SM, Melander O, Altshuler D, Merlini PA, Berzuini C, Bernardinelli L, Peyvandi F, Tubaro M, Celli P, Ferrario MM, Fetiveau R, Marziliano N, Casari G, Galli M, Ribichini F, Rossi M, Bernardi F, Zonzin P, Piazza A, Yee J, Friedlander Y, Marrugat J, Lucas G, Subirana I, Sala J, Ramos R, Meigs JB, Williams G, Nathan DM, MacRae CA, Havulinna AS, Berglund G, Hirschhorn JN, Asselta R, Duga S, Spreafico M, Daly MJ, Nemesh J, Korn JM, McCarroll SA, Surti A, Guiducci C, Gianniny L, Mirel D, Parkin M, Burtt N, Gabriel SB, Thompson JR, Braund PS, Wright BJ, Balmforth AJ, Ball SG, Hall AS, Wellcome Trust Case Control C, Linsel-Nitschke P, Lieb W, Ziegler A, Konig IR, Hengstenberg C, Fischer M, Stark K, Grosshennig A, Preuss M, Wichmann H-EE, Schreiber S, Ouwehand WH, Deloukas P, Scholz M, Cambien F, Li M, Chen Z, Wilensky R, Matthai W, Qasim A, Hakonarson HH, Devaney JM, Burnett MS, Pichard AD, Kent KM, Satler L, Lindsay JM, Waksman R, Knouff CW, Waterworth DM, Walker MC, Mooser V, Epstein SE, Scheffold T, Berger K, Huge A, Martinelli N, Olivieri O, Corrocher R, McKeown PP, Erdmann E, Konig IR, Holm H, Thorleifsson G, Thorsteinsdottir U, Stefansson K, Do R, Xie C, Siscovick DS, The CARDIoGRAMplusC4D Consortium, Studies TIC for BPG-WA, The CARDIoGRAMplusC4D Consortium, Preuss M, König IR, Thompson JR, Erdmann J, Absher D, Assimes TL, Blankenberg SS, Boerwinkle E, Chen L, Cupples LA, Hall AS, Halperin E, Hengstenberg C, Holm H, Laaksonen R, Li M, Marz W, McPherson R, Musunuru K, Nelson CP, Burnett MS, Epstein SE, O’Donnell CJ, Quertermous T, Rader DJ, Roberts R, Schillert A, Stefansson K, Stewart AFR, Thorleifsson G, Voight BF, Wells G a., Ziegler A, Kathiresan S, Reilly MP, Samani NJ, Schunkert H, Avenell A, Broom J, Brown TJ, Poobalan A, Aucott L, Stearns SC, Smith WCS, Jung RT, Campbell MK, Grant AM, Rakyan VK, Down TA, Balding DJ, Beck S, Schunkert H, König IR, Kathiresan S, Reilly MP, Assimes TL, Holm H, Preuss M, Stewart AFR, Barbalic M, Gieger C, Absher D, Aherrahrou Z, Allayee H, Altshuler D, Anand SS, Andersen K, Anderson JL, Ardissino D, Ball SG, Balmforth AJ, Barnes TA, Becker DM, Becker LC, Berger K, Bis JC, Boekholdt SM, Boerwinkle E, Braund PS, Brown MJ, Burnett MS, Buysschaert I, Carlquist JF, Chen L, Cichon S, Codd V, Davies RW, Dedoussis G, Dehghan A, Demissie S, Devaney JM, Diemert P, Do R, Doering A, Eifert S, Mokhtari NEE El, Ellis SG, Elosua R, Engert JC, Epstein SE, de Faire U, Fischer M, Folsom AR, Freyer J, Gigante B, Girelli D, Gretarsdottir S, Gudnason VV, Gulcher JR, Halperin E, Hammond N, Hazen SL, Hofman A, Horne BD, Illig T, Iribarren C, Jones GT, Jukema JW, Kaiser MA, Kaplan LM, Kastelein JJP, Khaw K-T, Knowles JW, Kolovou G, Kong A, Laaksonen R, Lambrechts D, Leander K, Lettre G, Li M, Lieb W, Loley C, Lotery AJ, Mannucci PM, Maouche S, Martinelli N, McKeown PP, Meisinger C, Meitinger T, Melander O, Merlini PA, Mooser V, Morgan T, Mühleisen TW, Muhlestein JB, Münzel T, Musunuru K, Nahrstaedt J, Nelson CP, Nöthen MM, Olivieri O, Patel RS, Patterson CC, Peters A, Peyvandi F, Qu L, Quyyumi AA, Rader DJ, Rallidis LS, Rice C, Rosendaal FR, Rubin D, Salomaa V, Sampietro ML, Sandhu MS, Schadt E, Schäfer A, Schillert A, Schreiber S, Schrezenmeir J, Schwartz SM, Siscovick DS, Sivananthan M, Sivapalaratnam S, Smith A, Smith TB, Snoep JD, Soranzo N, Spertus JA, Stark K, Stirrups KK, Stoll M, Tang WHW, Tennstedt S, Thorgeirsson G, Thorleifsson G, Tomaszewski M, Uitterlinden AG, van Rij AM, Voight BF, Wareham NJN, Wells G a., Wichmann H-EE, Wild PS, Willenborg C, Witteman JCM, Wright BJ, Ye S, Zeller T, Ziegler A, Cambien F, Goodall AH, Cupples LA, Quertermous T, März W, Hengstenberg C, Blankenberg SS, Ouwehand WH, Hall AS, Deloukas P, Thompson JR, Stefansson K, Roberts R, Thorsteinsdottir U, O’Donnell CJ, McPherson R, Erdmann J, Samani NJ, Sharma P, Garg G, Kumar A, Mohammad F, Ramesh S, Slatkin M, Myocardial T, Genetics I, Investigators C, Udali S, Guarini P, Moruzzi S, Choi S, Friso S, Schübeler D, Setten J Van, Isgum I, Smolonska J, Ripke S, Jong PA De, Oudkerk M, Koning H De, Lammers JJ, Zanen P, Groen HJM, Boezen HM, Postma DS, Wijmenga C, Viergever MA, Th WP, Bakker PIW De (2015) Genome-wide association of early-onset myocardial infarction with single nucleotide polymorphisms and copy number variants. Nat Genet 478:103–109. doi: 10.1038/nrg3000.Epigenome-Wide

2. Burton PR, Clayton DG, Cardon LR, Craddock N, Deloukas P, Duncanson A, Kwiatkowski DP, McCarthy MI, Ouwehand WH, Samani NJ, Todd JA, Donnelly P, Barrett JC, Davison D, Easton D, Evans D, Leung HT, Marchini JL, Morris AP, Spencer CCA, Tobin MD, Attwood AP, Boorman JP, Cant B, Everson U, Hussey JM, Jolley JD, Knight AS, Koch K, Meech E, Nutland S, Prowse C V., Stevens HE, Taylor NC, Walters GR, Walker NM, Watkins NA, Winzer T, Jones RW, McArdle WL, Ring SM, Strachan DP, Pembrey M, Breen G, St. Clair D, Caesar S, Gordon-Smith K, Jones L, Fraser C, Green EK, Grozeva D, Hamshere ML, Holmans PA, Jones IR, Kirov G, Moskvina V, Nikolov I, O’Donovan MC, Owen MJ, Collier DA, Elkin A, Farmer A, Williamson R, McGuffin P, Young AH, Ferrier IN, Ball SG, Balmforth AJ, Barrett JH, Bishop DT, Iles MM, Maqbool A, Yuldasheva N, Hall AS, Braund PS, Dixon RJ, Mangino M, Stevens S, Thompson JR, Bredin F, Tremelling M, Parkes M, Drummond H, Lees CW, Nimmo ER, Satsangi J, Fisher SA, Forbes A, Lewis CM, Onnie CM, Prescott NJ, Sanderson J, Mathew CG, Barbour J, Mohiuddin MK, Todhunter CE, Mansfield JC, Ahmad T, Cummings FR, Jewell DP, Webster J, Brown MJ, Lathrop GM, Connell J, Dominiczak A, Braga Marcano CA, Burke B, Dobson R, Gungadoo J, Lee KL, Munroe PB, Newhouse SJ, Onipinla A, Wallace C, Xue M, Caulfield M, Farrall M, Barton A, Bruce IN, Donovan H, Eyre S, Gilbert PD, Hider SL, Hinks AM, John SL, Potter C, Silman AJ, Symmons DPM, Thomson W, Worthington J, Dunger DB, Widmer B, Frayling TM, Freathy RM, Lango H, Perry JRB, Shields BM, Weedon MN, Hattersley AT, Hitman GA, Walker M, Elliott KS, Groves CJ, Lindgren CM, Rayner NW, Timpson NJ, Zeggini E, Newport M, Sirugo G, Lyons E, Vannberg F, Hill AVS, Bradbury LA, Farrar C, Pointon JJ, Wordsworth P, Brown MA, Franklyn JA, Heward JM, Simmonds MJ, Gough SCL, Seal S, Stratton MR, Rahman N, Ban SM, Goris A, Sawcer SJ, Compston A, Conway D, Jallow M, Rockett KA, Bumpstead SJ, Chaney A, Downes K, Ghori MJR, Gwilliam R, Hunt SE, Inouye M, Keniry A, King E, McGinnis R, Potter S, Ravindrarajah R, Whittaker P, Widden C, Withers D, Cardin NJ, Ferreira T, Pereira-Gale J, Hallgrimsdóttir IB, Howie BN, Spencer CCA, Su Z, Teo YY, Vukcevic D, Bentley D, Compston A (2007) Genome-wide association study of 14,000 cases of seven common diseases and 3,000 shared controls. Nature 447:661–678. doi: 10.1038/nature05911

3. Bycroft C, Freeman C, Petkova D, Band G, Elliott LT, Sharp K, Motyer A, Vukcevic D, Delaneau O, O’Connell J, Cortes A, Welsh S, Young A, Effingham M, McVean G, Leslie S, Allen N, Donnelly P, Marchini J (2018) The UK Biobank resource with deep phenotyping and genomic data. Nature 562:203–209. doi: 10.1038/s41586-018-0579-z

4. Erdmann J, Großhennig A, Braund PS, König IR, Hengstenberg C, Hall AS, Linsel-Nitschke P, Kathiresan S, Wright B, Trégouët DA, Cambien F, Bruse P, Aherrahrou Z, Wagner AK, Stark K, Schwartz SM, Salomaa V, Elosua R, Melander O, Voight BF, O’Donnell CJ, Peltonen L, Siscovick DS, Altshuler D, Merlini PA, Peyvandi F, Bernardinelli L, Ardissino D, Schillert A, Blankenberg S, Zeller T, Wild P, Schwarz DF, Tiret L, Perret C, Schreiber S, Mokhtari NE El, Schäfer A, März W, Renner W, Bugert P, Klüter H, Schrezenmeir J, Rubin D, Ball SG, Balmforth AJ, Wichmann HE, Meitinger T, Fischer M, Meisinger C, Baumert J, Peters A, Ouwehand WH, Deloukas P, Thompson JR, Ziegler A, Samani NJ, Schunkert H (2009) New susceptibility locus for coronary artery disease on chromosome 3q22.3. Nat Genet 41:280–282. doi: 10.1038/ng.307

5. Erdmann J, Willenborg C, Nahrstaedt J, Preuss M, Konig IR, Baumert J, Linsel-Nitschke P, Gieger C, Tennstedt S, Belcredi P, Aherrahrou Z, Klopp N, Loley C, Stark K, Hengstenberg C, Bruse P, Freyer J, Wagner AK, Medack A, Lieb W, Grosshennig A, Sager HB, Reinhardt A, Schafer A, Schreiber S, El Mokhtari NE, Raaz-Schrauder D, Illig T, Garlichs CD, Ekici AB, Reis A, Schrezenmeir J, Rubin D, Ziegler A, Wichmann H-E, Doering A, Meisinger C, Meitinger T, Peters A, Schunkert H (2011) Genome-wide association study identifies a new locus for coronary artery disease on chromosome 10p11.23. Eur Heart J 32:158–168. doi: 10.1093/eurheartj/ehq405

6. Franzén O, Ermel R, Cohain A, Akers NK, Di Narzo A, Talukdar HA, Foroughi-Asl H, Giambartolomei C, Fullard JF, Sukhavasi K, Köks S, Gan LM, Giannarelli C, Kovacic JC, Betsholtz C, Losic B, Michoel T, Hao K, Roussos P, Skogsberg J, Ruusalepp A, Schadt EE, Björkegren JLM (2016) Cardiometabolic risk loci share downstream cis- and trans-gene regulation across tissues and diseases. Science (80- ) 353:827–830. doi: 10.1126/science.aad6970

7. GTEx Consortium (2013) The Genotype-Tissue Expression (GTEx) project. Nat Genet 45:580–5. doi: 10.1038/ng.2653

8. Huckins LM, Dobbyn A, Ruderfer DM, Hoffman G, Wang W, Pardiñas AF, Rajagopal VM, Als TD, T. Nguyen H, Girdhar K, Boocock J, Roussos P, Fromer M, Kramer R, Domenici E, Gamazon ER, Purcell S, Johnson JS, Shah HR, Klein LL, Dang KK, Logsdon BA, Mahajan MC, Mangravite LM, Toyoshiba H, Gur RE, Hahn CG, Schadt E, Lewis DA, Haroutunian V, Peters MA, Lipska BK, Buxbaum JD, Hirai K, Perumal TM, Essioux L, Ripke S, Neale BM, Corvin A, Walters JTR, Farh KH, Holmans PA, Lee P, Bulik-Sullivan B, Collier DA, Huang H, Pers TH, Agartz I, Agerbo E, Albus M, Alexander M, Amin F, Bacanu SA, Begemann M, Belliveau RA, Bene J, Bergen SE, Bevilacqua E, Bigdeli TB, Black DW, Bruggeman R, Buccola NG, Buckner RL, Byerley W, Cahn W, Cai G, Campion D, Cantor RM, Carr VJ, Carrera N, Catts S V., Chambert KD, Chan RCK, Chen RYL, Chen EYH, Cheng W, Cheung EFC, Chong SA, Cloninger CR, Cohen D, Cohen N, Cormican P, Craddock N, Crowley JJ, Curtis D, Davidson M, Davis KL, Degenhardt F, Del Favero J, Demontis D, Dikeos D, Dinan T, Djurovic S, Donohoe G, Drapeau E, Duan J, Dudbridge F, Durmishi N, Eichhammer P, Eriksson J, Escott-Price V, Fanous AH, Farrell MS, Frank J, Franke L, Freedman R, Freimer NB, Friedl M, Friedman JI, Fromer M, Genovese G, Georgieva L, Giegling I, Giusti-Rodríguez P, Godard S, Goldstein JI, Golimbet V, Gopal S, Gratten J, de Haan L, Hammer C, Hamshere ML, Hansen M, Hansen T, Hartmann AM, Henskens FA, Herms S, Hirschhorn JN, Hoffmann P, Hofman A, Hollegaard M V., Hougaard DM, Ikeda M, Joa I, Julia A, Kahn RS, Kalaydjieva L, Karachanak-Yankova S, Karjalainen J, Kavanagh D, Keller MC, Kennedy JL, Khrunin A, Kim Y, Klovins J, Knowles JA, Konte B, Kucinskas V, Kucinskiene ZA, Kuzelova-Ptackova H, Kahler AK, Laurent C, Keong JLC, Lee SH, Legge SE, Lerer B, Li M, Li T, Liang KY, Lieberman J, Limborska S, Loughland CM, Lubinski J, Lonnqvist J, Macek M, Magnusson PKE, Maher BS, Maier W, Mallet J, Marsal S, Mattheisen M, Mattingsdal M, McCarley RW, McDonald C, McIntosh AM, Meier S, Meijer CJ, Melegh B, Melle I, Mesholam-Gately RI, Metspalu A, Michie PT, Milani L, Milanova V, Mokrab Y, Morris DW, Mors O, Murphy KC, Murray RM, Myin-Germeys I, Muller-Myhsok B, Nelis M, Nenadic I, Nertney DA, Nestadt G, Nicodemus KK, Nikitina-Zake L, Nisenbaum L, Nordin A, O’Callaghan E, O’Dushlaine C, O’Neill FA, Oh SY, Olincy A, Olsen L, Van Os J, Pantelis C, Papadimitriou GN, Papiol S, Parkhomenko E, Pato MT, Paunio T, Pejovic-Milovancevic M, Perkins DO, Pietiläinen O, Pimm J, Pocklington AJ, Powell J, Price A, Pulver AE, Purcell SM, Quested D, Rasmussen HB, Reichenberg A, Reimers MA, Richards AL, Roffman JL, Salomaa V, Sanders AR, Schall U, Schubert CR, Schulze TG, Schwab SG, Scolnick EM, Scott RJ, Seidman LJ, Shi J, Sigurdsson E, Silagadze T, Silverman JM, Sim K, Slominsky P, Smoller JW, So HC, Spencer CCA, Stahl EA, Stefansson H, Steinberg S, Stogmann E, Straub RE, Strengman E, Strohmaier J, Stroup TS, Subramaniam M, Suvisaari J, Svrakic DM, Szatkiewicz JP, Soderman E, Thirumalai S, Toncheva D, Tosato S, Veijola J, Waddington J, Walsh D, Wang D, Wang Q, Webb BT, Weiser M, Wildenauer DB, Williams NM, Williams S, Witt SH, Wolen AR, Wong EHM, Wormley BK, Xi HS, Zai CC, Zheng X, Zimprich F, Wray NR, Stefansson K, Visscher PM, Adolfsson R, Andreassen OA, Blackwood DHR, Bramon E, Børglum AD, Cichon S, Darvasi A, Ehrenreich H, Esko T, Gejman P V., Gill M, Gurling H, Hultman CM, Iwata N, Jablensky A V., Jonsson EG, Kendler KS, Kirov G, Knight J, Lencz T, Levinson DF, Li QS, Liu J, Malhotra AK, McCarroll SA, McQuillin A, Moran JL, Mortensen PB, Mowry BJ, Nothen MM, Ophoff RA, Owen MJ, Palotie A, Pato CN, Petryshen TL, Posthuma D, Rietschel M, Riley BP, Rujescu D, Sham PC, Sklar P, Clair DS, Weinberger DR, Wendland JR, Werge T, Daly MJ, Sullivan PF, O’Donovan MC, Rajagopal VM, Grove J, Mortensen PB, Pedersen CB, Pedersen MG, Nordentoft M, Bybjerg-Grauholm J, Bækvad-Hansen M, Hansen CS, Sullivan P, Devlin B, Sieberts SK, Cox NJ, Im HK (2019) Gene expression imputation across multiple brain regions provides insights into schizophrenia risk. Nat Genet 51:659–674. doi: 10.1038/s41588-019-0364-4

9. Li L, Pang S, Zeng L, Güldener U, Schunkert H (2021) Genetically determined intelligence and coronary artery disease risk. Clin Res Cardiol 110:211–219. doi: 10.1007/s00392-020-01721-x

10. Nelson CP, Goel A, Butterworth AS, Kanoni S, Webb TR, Marouli E, Zeng L, Ntalla I, Lai FY, Hopewell JC, Giannakopoulou O, Jiang T, Hamby SE, Di Angelantonio E, Assimes TL, Bottinger EP, Chambers JC, Clarke R, Palmer CNA, Cubbon RM, Ellinor P, Ermel R, Evangelou E, Franks PW, Grace C, Gu D, Hingorani AD, Howson JMM, Ingelsson E, Kastrati A, Kessler T, Kyriakou T, Lehtimäki T, Lu X, Lu Y, März W, McPherson R, Metspalu A, Pujades-Rodriguez M, Ruusalepp A, Schadt EE, Schmidt AF, Sweeting MJ, Zalloua PA, Alghalayini K, Keavney BD, Kooner JS, Loos RJF, Patel RS, Rutter MK, Tomaszewski M, Tzoulaki I, Zeggini E, Erdmann J, Dedoussis G, Björkegren JLM, Schunkert H, Farrall M, Danesh J, Samani NJ, Watkins H, Deloukas P (2017) Association analyses based on false discovery rate implicate new loci for coronary artery disease. Nat Genet 49:1385–1391. doi: 10.1038/ng.3913

11. Nikpay M, Goel A, Won HH, Hall LM, Willenborg C, Kanoni S, Saleheen D, Kyriakou T, Nelson CP, CHopewell J, Webb TR, Zeng L, Dehghan A, Alver M, MArmasu S, Auro K, Bjonnes A, Chasman DI, Chen S, Ford I, Franceschini N, Gieger C, Grace C, Gustafsson S, Huang J, Hwang SJ, Kim YK, Kleber ME, Lau KW, Lu X, Lu Y, Lyytikäinen LP, Mihailov E, Morrison AC, Pervjakova N, Qu L, Rose LM, Salfati E, Saxena R, Scholz M, Smith A V., Tikkanen E, Uitterlinden A, Yang X, Zhang W, Zhao W, De Andrade M, De Vries PS, Van Zuydam NR, Anand SS, Bertram L, Beutner F, Dedoussis G, Frossard P, Gauguier D, Goodall AH, Gottesman O, Haber M, Han BG, Huang J, Jalilzadeh S, Kessler T, König IR, Lannfelt L, Lieb W, Lind L, MLindgren C, Lokki ML, Magnusson PK, Mallick NH, Mehra N, Meitinger T, Memon FUR, Morris AP, Nieminen MS, Pedersen NL, Peters A, Rallidis LS, Rasheed A, Samuel M, Shah SH, Sinisalo J, EStirrups K, Trompet S, Wang L, Zaman KS, Ardissino D, Boerwinkle E, Borecki IB, Bottinger EP, Buring JE, Chambers JC, Collins R, Cupples L, Danesh J, Demuth I, Elosua R, Epstein SE, Esko T, Feitosa MF, Franco OH, Franzosi MG, Granger CB, Gu D, Gudnason V, SHall A, Hamsten A, Harris TB, LHazen S, Hengstenberg C, Hofman A, Ingelsson E, Iribarren C, Jukema JW, Karhunen PJ, Kim BJ, Kooner JS, Kullo IJ, Lehtimäki T, Loos RJF, Melander O, Metspalu A, März W, Palmer CN, Perola M, Quertermous T, Rader DJ, Ridker PM, Ripatti S, Roberts R, Salomaa V, Sanghera DK, Schwartz SM, Seedorf U, Stewart AF, Stott DJ, Thiery J, Zalloua PA, O’Donnell CJ, Reilly MP, Assimes TL, Thompson JR, Erdmann J, Clarke R, Watkins H, Kathiresan S, McPherson R, Deloukas P, Schunkert H, Samani NJ, Farrall M (2015) A comprehensive 1000 Genomes-based genome-wide association meta-analysis of coronary artery disease. Nat Genet 47:1121–1130. doi: 10.1038/ng.3396

12. Samani NJ, Erdmann J, Hall AS, Hengstenberg C, Mangino M, Mayer B, Dixon RJ, Meitinger T, Braund P, Wichmann H-E, Barrett JH, König IR, Stevens SE, Szymczak S, Tregouet D-A, Iles MM, Pahlke F, Pollard H, Lieb W, Cambien F, Fischer M, Ouwehand W, Blankenberg S, Balmforth AJ, Baessler A, Ball SG, Strom TM, Brænne I, Gieger C, Deloukas P, Tobin MD, Ziegler A, Thompson JR, Schunkert H (2007) Genomewide Association Analysis of Coronary Artery Disease. N Engl J Med 357:443–453. doi: 10.1056/NEJMoa072366

13. Stitziel NO, Won HH, Morrison AC, Peloso GM, Do R, Lange LA, Fontanillas P, Gupta N, Duga S, Goel A, Farrall M, Saleheen D, Ferrario P, König I, Asselta R, Merlini PA, Marziliano N, Notarangelo MF, Schick U, Auer P, Assimes TL, Reilly M, Wilensky R, Rader DJ, Kees Hovingh G, Meitinger T, Kessler T, Kastrati A, Laugwitz KL, Siscovick D, Rotter JI, Hazen SL, Tracy R, Cresci S, Spertus J, Jackson R, Schwartz SM, Natarajan P, Crosby J, Muzny D, Ballantyne C, Rich SS, O’Donnell CJ, Abecasis G, Sunyaev S, Nickerson DA, Buring JE, Ridker PM, Chasman DI, Austin E, Ye Z, Kullo IJ, Weeke PE, Shaffer CM, Bastarache LA, Denny JC, Roden DM, Palmer C, Deloukas P, Lin DY, Tang ZZ, Erdmann J, Schunkert H, Danesh J, Marrugat J, Elosua R, Ardissino D, McPherson R, Watkins H, Reiner AP, Wilson JG, Altshuler D, Gibbs RA, Lander ES, Boerwinkle E, Gabriel S, Kathiresan S (2014) Inactivating mutations in NPC1L1 and protection from coronary heart disease. N Engl J Med 371:2072–2082. doi: 10.1056/NEJMoa1405386

14. Winkelmann BR, März W, Boehm BO, Zotz R, Hager J, Hellstern P, Senges J (2001) Rationale and design of the LURIC study - A resource for functional genomics, pharmacogenomics and long-term prognosis of cardiovascular disease. Pharmacogenomics 2. doi: 10.1517/14622416.2.1.s1

15. Wu L, Shi W, Long J, Guo X, Michailidou K, Beesley J, Bolla MK, Shu XO, Lu Y, Cai Q, Al-Ejeh F, Rozali E, Wang Q, Dennis J, Li B, Zeng C, Feng H, Gusev A, Barfield RT, Andrulis IL, Anton-Culver H, Arndt V, Aronson KJ, Auer PL, Barrdahl M, Baynes C, Beckmann MW, Benitez J, Bermisheva M, Blomqvist C, Bogdanova N V., Bojesen SE, Brauch H, Brenner H, Brinton L, Broberg P, Brucker SY, Burwinkel B, Caldés T, Canzian F, Carter BD, Castelao JE, Chang-Claude J, Chen X, Cheng TYD, Christiansen H, Clarke CL, Collée M, Cornelissen S, Couch FJ, Cox D, Cox A, Cross SS, Cunningham JM, Czene K, Daly MB, Devilee P, Doheny KF, Dörk T, Dos-Santos-Silva I, Dumont M, Dwek M, Eccles DM, Eilber U, Eliassen AH, Engel C, Eriksson M, Fachal L, Fasching PA, Figueroa J, Flesch-Janys D, Fletcher O, Flyger H, Fritschi L, Gabrielson M, Gago-Dominguez M, Gapstur SM, García-Closas M, Gaudet MM, Ghoussaini M, Giles GG, Goldberg MS, Goldgar DE, González-Neira A, Guénel P, Hahnen E, Haiman CA, Håkansson N, Hall P, Hallberg E, Hamann U, Harrington P, Hein A, Hicks B, Hillemanns P, Hollestelle A, Hoover RN, Hopper JL, Huang G, Humphreys K, Hunter DJ, Jakubowska A, Janni W, John EM, Johnson N, Jones K, Jones ME, Jung A, Kaaks R, Kerin MJ, Khusnutdinova E, Kosma VM, Kristensen VN, Lambrechts D, Le Marchand L, Li J, Lindström S, Lissowska J, Lo WY, Loibl S, Lubinski J, Luccarini C, Lux MP, MacInnis RJ, Maishman T, Kostovska IM, Mannermaa A, Manson JAE, Margolin S, Mavroudis D, Meijers-Heijboer H, Meindl A, Menon U, Meyer J, Mulligan AM, Neuhausen SL, Nevanlinna H, Neven P, Nielsen SF, Nordestgaard BG, Olopade OI, Olson JE, Olsson H, Peterlongo P, Peto J, Plaseska-Karanfilska D, Prentice R, Presneau N, Pylkäs K, Rack B, Radice P, Rahman N, Rennert G, Rennert HS, Rhenius V, Romero A, Romm J, Rudolph A, Saloustros E, Sandler DP, Sawyer EJ, Schmidt MK, Schmutzler RK, Schneeweiss A, Scott RJ, Scott CG, Seal S, Shah M, Shrubsole MJ, Smeets A, Southey MC, Spinelli JJ, Stone J, Surowy H, Swerdlow AJ, Tamimi RM, Tapper W, Taylor JA, Terry MB, Tessier DC, Thomas A, Thöne K, Tollenaar RAEM, Torres D, Truong T, Untch M, Vachon C, Van Den Berg D, Vincent D, Waisfisz Q, Weinberg CR, Wendt C, Whittemore AS, Wildiers H, Willett WC, Winqvist R, Wolk A, Xia L, Yang XR, Ziogas A, Ziv E, Dunning AM, Pharoah PDP, Simard J, Milne RL, Edwards SL, Kraft P, Easton DF, Chenevix-Trench G, Zheng W (2018) A transcriptome-wide association study of 229,000 women identifies new candidate susceptibility genes for breast cancer. Nat Genet 50:968–978. doi: 10.1038/s41588-018-0132-x

16. Zhang W, Voloudakis G, Rajagopal VM, Readhead B, Dudley JT, Schadt EE, Björkegren JLM, Kim Y, Fullard JF, Hoffman GE, Roussos P (2019) Integrative transcriptome imputation reveals tissue-specific and shared biological mechanisms mediating susceptibility to complex traits. Nat Commun 10:1–13. doi: 10.1038/s41467-019-11874-7

# Supplementary Tables

Supplementary Table 1. Statistics of nine tissues' predictive models.

Supplementary Table 2. SNP priors of COR and TIB tissues.

Supplementary Table 3. 11 Genotype cohorts.

Supplementary Table 4. 114 TWAS genes list.

Supplementary Table 5. 53 TWAS genes have strong evidence of colocalized signals between GWAS and eQTL (PP4 > 0.55).

Supplementary Table 6. 96 known and 18 novel genes annotated by GWAS risk loci of CAD.

Supplementary Table 7. TWAS genes are enriched to CAD or related risk traits based on DisGeNET.

Supplementary Table 8. Pathways enriched by TWAS genes.

Supplementary Table 9. Association of TWAS genes' damaging mutation with CAD and its binary risk traits.

Supplementary Table 10. Association of TWAS genes' damaging variants with quantitative risk traits of CAD.

Supplementary Table 11. Lead variants resided in the regions of novel genes were associated with lipid traits in human genotype data.

Supplementary Table 12. Expression-trait association statistics in mouse atherosclerosis model from HMDP.

Supplementary Table 13. Functional summary of 18 novel genes.

Supplementary Table 14. Differentially expressed genes in *RGS19*-KO hepatocytes.

Supplementary Table 15. Differentially expressed genes in *KPTN*-KO hepatocytes.

# Supplementary Figures

**
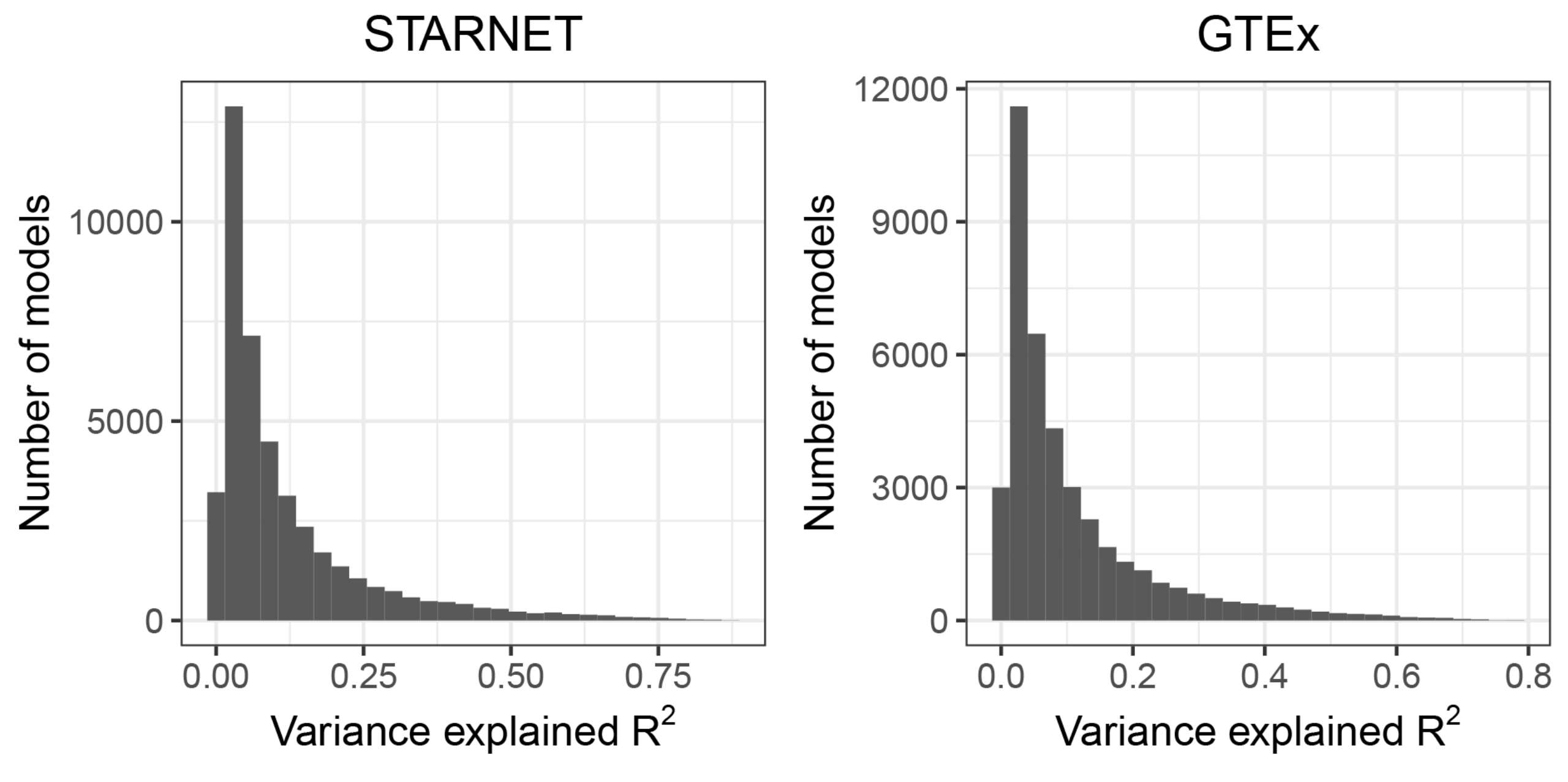
**

**Supplementary Fig. 1** Distribution of cross-validation prediction accuracy of expression (R^2^) for models trained from Stockholm-Tartu Atherosclerosis Reverse Network Engineering Task (STARNET) (a) and the Genotype-Tissue Expression (GTEx) (b).

**a**

**b**


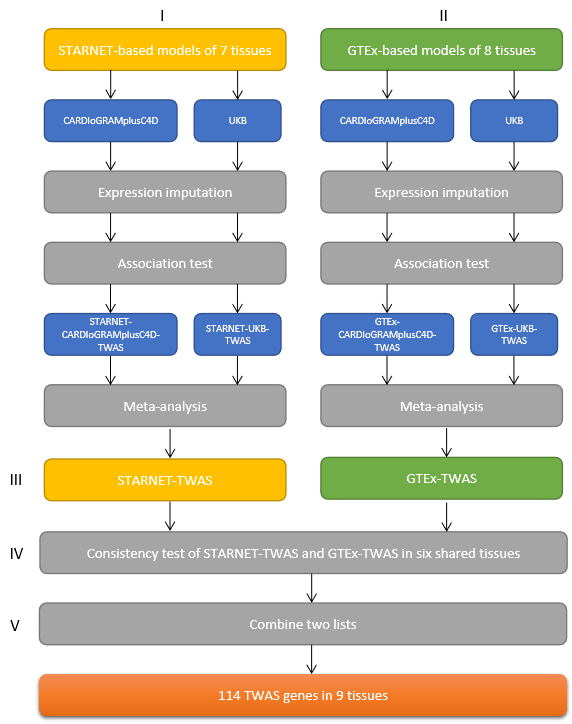


**Supplementary Fig. 2** Cross validation of transcriptome-wide association analyses. The prediction models were trained from the Stockholm-Tartu Atherosclerosis Reverse Network Engineering Task panel (STARNET) (I) and the Genotype-Tissue Expression panel (GTEx) (II). The eleven genotype cohorts were used as discovery (CARDIoGRAMplusC4D) and replication set (UK Biobank, UKB). Within STARNET-based models of 7 tissues (I), 49 genes were Bonferroni-significant in the discovery set and 12 were replicated in the replication set (P<3.85e-6). Within GTEx-based models of 8 tissues, 35 genes were Bonferroni-significant in the discover set and 14 were replicated in the replication set (P<3.85e-6). To increase power, we performed a meta-analysis on eleven cohorts for each tissue within panels and obtained STARNET- and GTEx-TWAS statistics (III). Through comparation of TWAS statistics in six tissues shared between two panels, we observed consistency and complementarity between panels (Supplementary Fig. 4-5). Therefore, we combined two list and finalized on 114 TWAS genes of nine tissues (V).


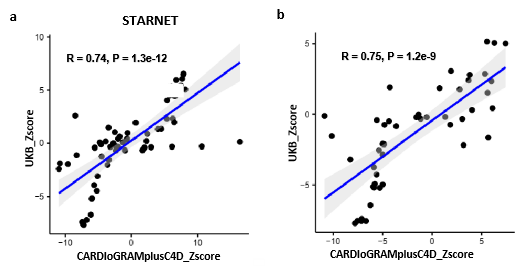


**Supplementary Fig. 3** Replicability of TWAS results within two panels. a) Replicability of STARNET-based models. b) Reproducibility of GTEx-based models. Ten CARDIoGRAMplusC4D cohorts were used as the testing set, genotypes from UK Biobank (UKB) were the validating set. STARNET, the Stockholm-Tartu Atherosclerosis Reverse Network Engineering panel; GTEx, the Genotype-Tissue Expression panel; Zscore, z-score.


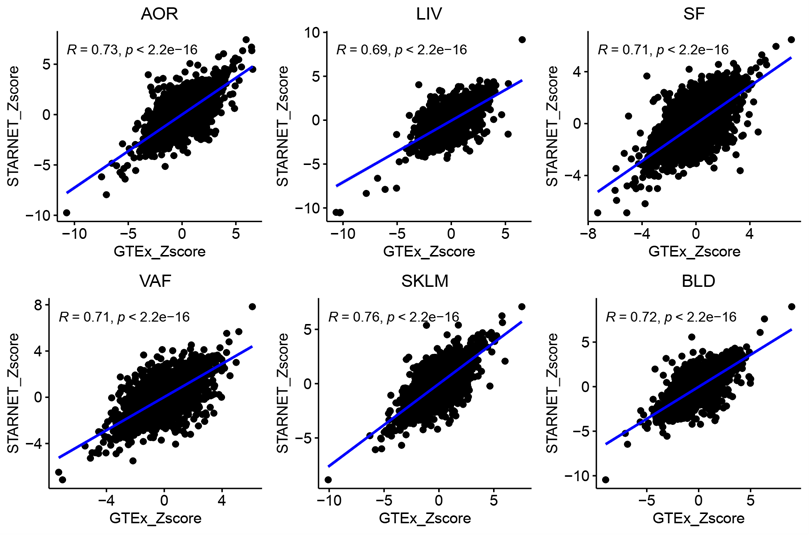


**Supplementary Fig.** 4 Associations of predicted expressions with CAD are consistent across tissues between STARNET- and GTEx-based models. AOR, aorta; LIV, liver; SF, subcutaneous fat; VAF, visceral abdominal fat; SKLM, skeletal muscle; BLD, blood; STARNET, the Stockholm-Tartu Atherosclerosis Reverse Network Engineering panel; G TEx, the Genotype-Tissue Expression panel; Zscore, z-score.


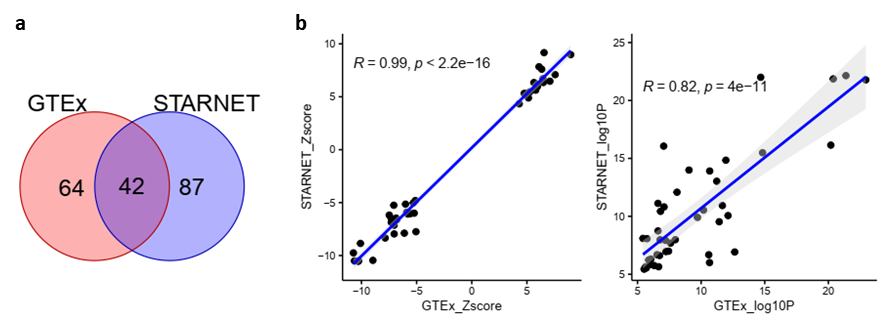


**Supplementary Fig. 5** Comparison of TWAS results between two reference models. (A) Venn diagram of transcriptome-wide significant gene-tissue pairs based on the two reference models. There are 42 overlapping gene-tissue pairs (34 genes). (B) The effect sizes (left) and P values (right) of overlapping genes were consistent between the two reference-based models. STARNET, the Stockholm-Tartu Atherosclerosis Reverse Network Engineering panel; GTEx, the Genotype-Tissue Expression panel; Zscore, z-score.


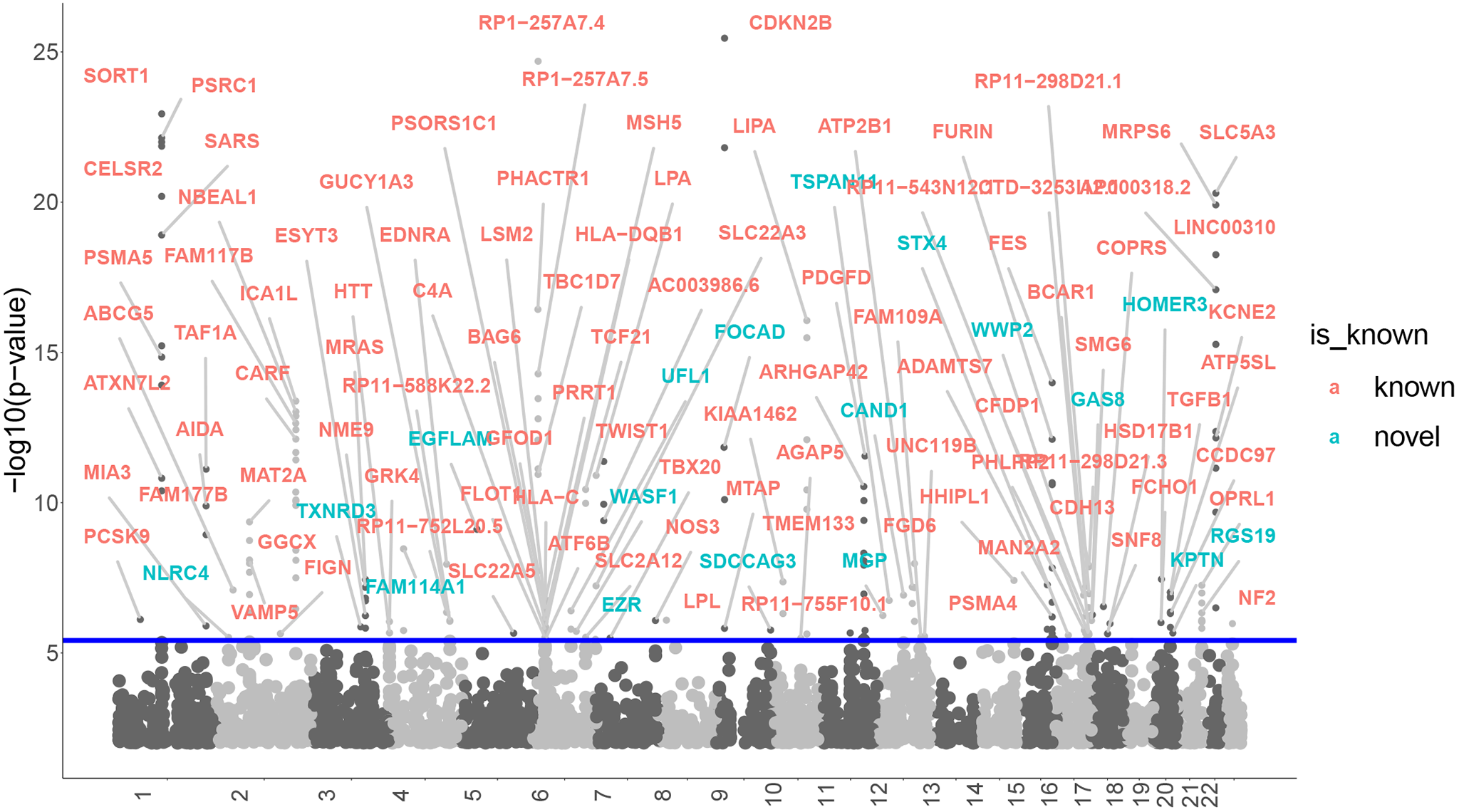


**Supplementary Fig. 6** Manhattan plot of TWAS results of CAD. 114 CAD TWAS genes are highlighted. The blue line marks *P*=3.85e-6. Each point corresponds to an association test between a gene-tissue pair. TWAS genes residing in genome-wide significant loci were defined as known (red writing), otherwise defined as novel (blue writing).


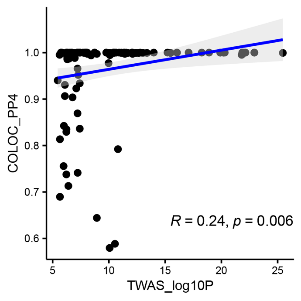


**Supplementary Fig. 7** Positive correlation between TWAS and colocalization statistics. The log10(P) statistics of TWAS genes were positively correlated with PP4 (the posterior probabilities) statistics of colocalization (COLOC) analysis. Most TWAS genes have shared casual variants between GWAS (genome-wide association study) signals and eQTL (expression quantitative trait loci) signals as their PP4 approached to 1.


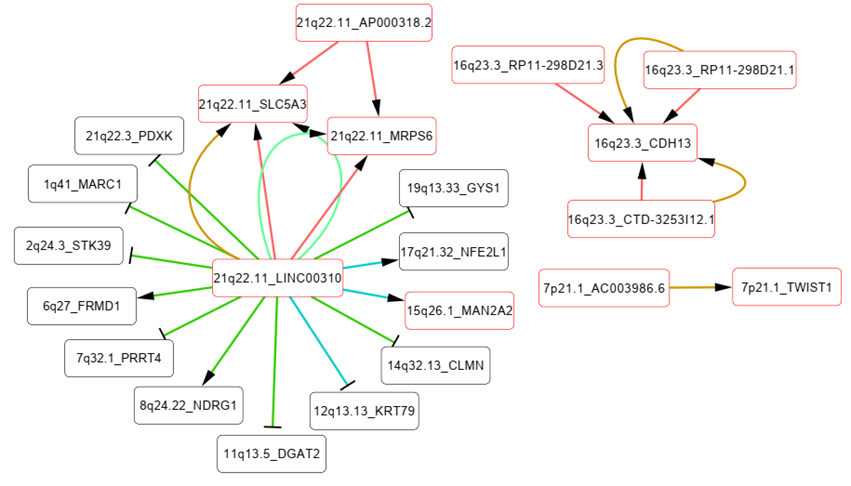


**Supplementary Fig. 8** Co-expression network related to lncRNA genes. Coding genes with co-expression relationship with TWAS lncRNA genes are linked by arrow or T-line. Arrow suggests positive co-expression, and T-line suggests negative. TWAS genes are shown in red frame. Tissues of gene co-expression are showed in difference edge colors as indicated. AOR, aorta; MAM mammary artery; BLD, blood; LIV, liver; SF, subcutaneous fat; VAF, visceral abdominal fat; SKLM, skeletal muscle.


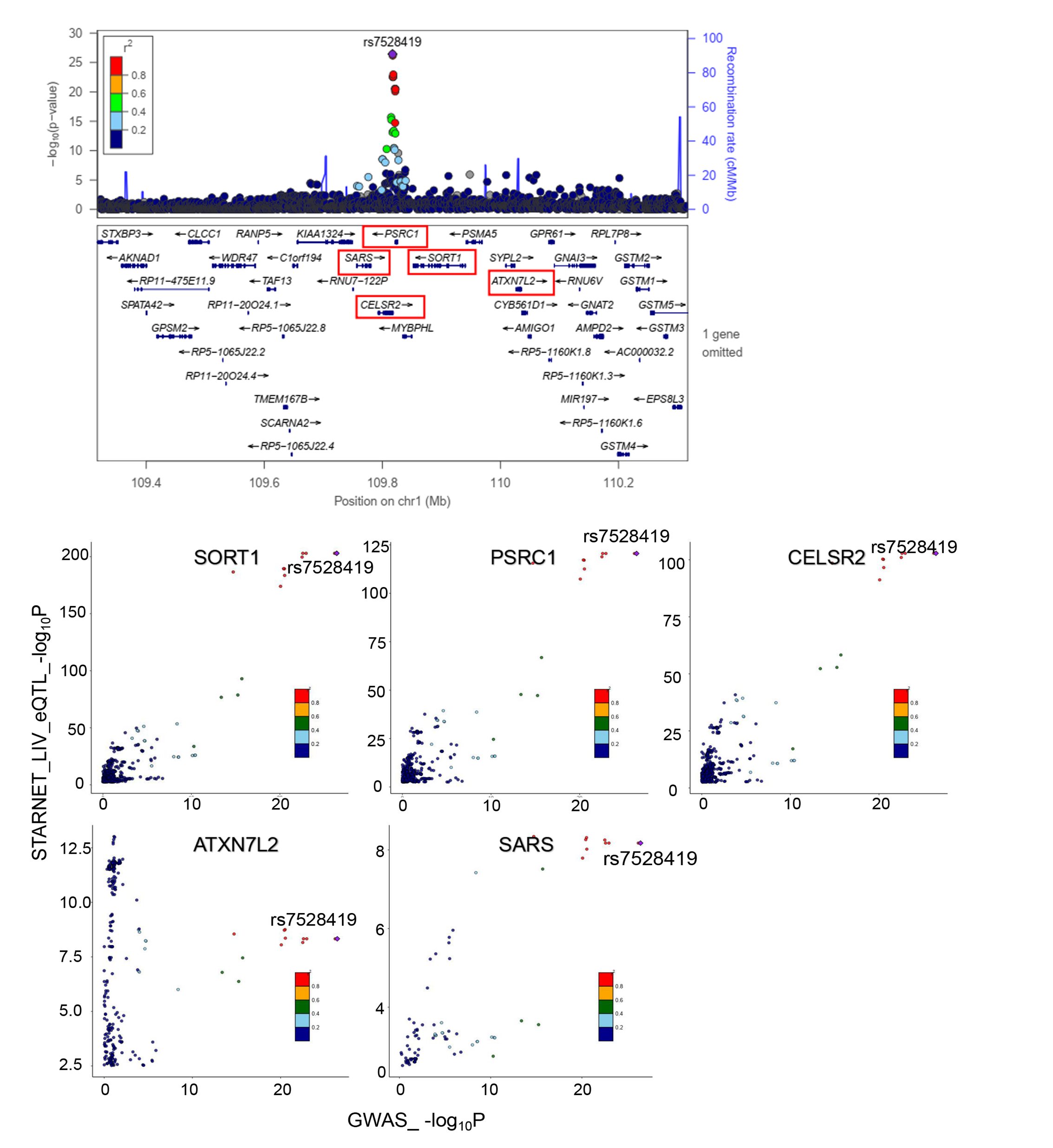


**Supplementary Fig. 9** Colocalization signals in liver tissue at 1p13.3. The red-framed genes in locuszoom plot are transcriptome-wide significant genes. LIV, liver; GWAS, genome-wide association study; STARNET, the Stockholm-Tartu Atherosclerosis Reverse Network Engineering panel; eQTL, expression quantitative trait loci.

**
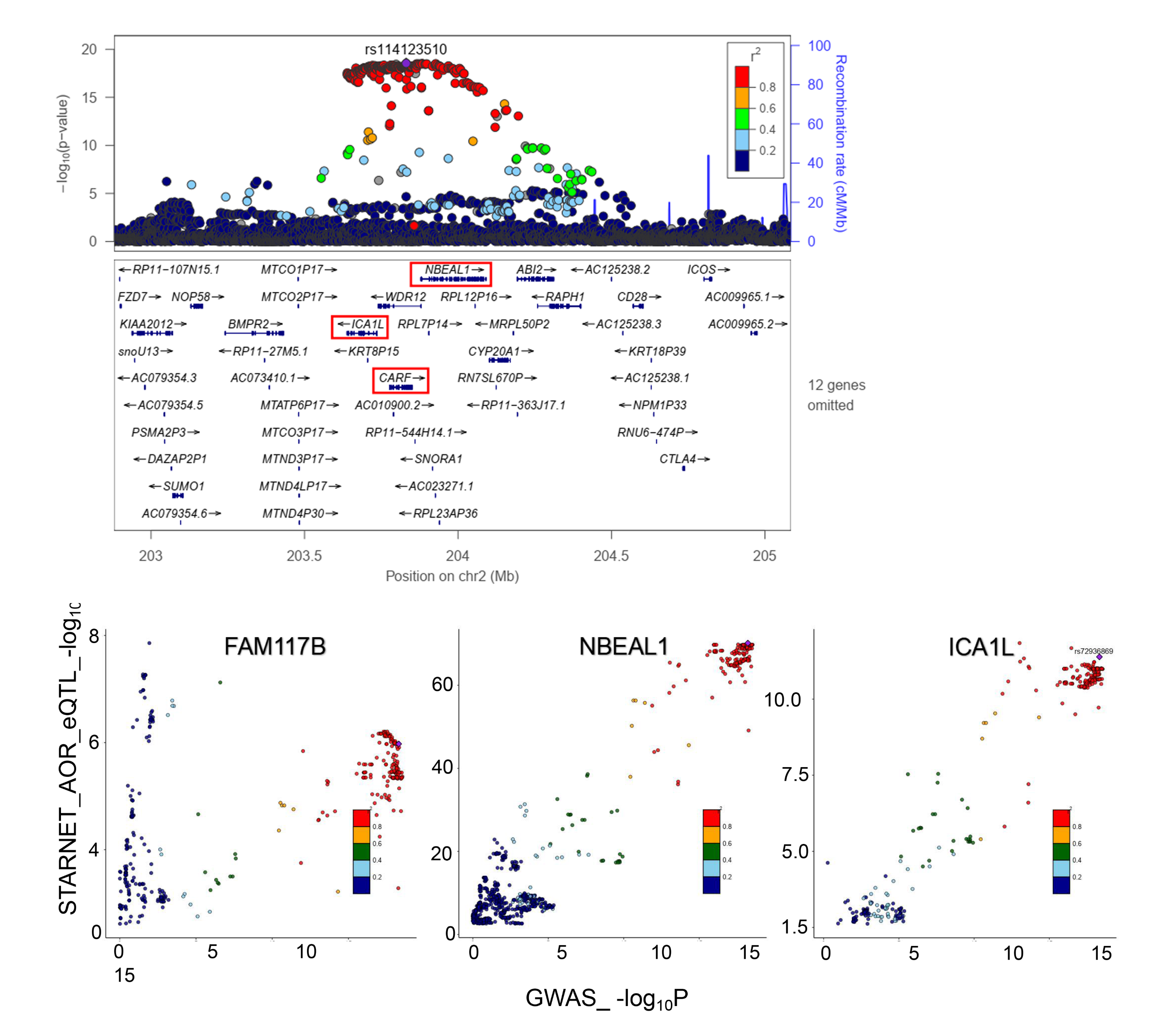
**

**Supplementary Fig. 10** Colocalization signals in aorta tissue at 2p33.2. The red-framed genes in locuszoom plot are transcriptome-wide significant genes. AOR, aorta; GWAS, genome-wide association study; STARNET, the Stockholm-Tartu Atherosclerosis Reverse Network Engineering panel; eQTL, expression quantitative trait loci.


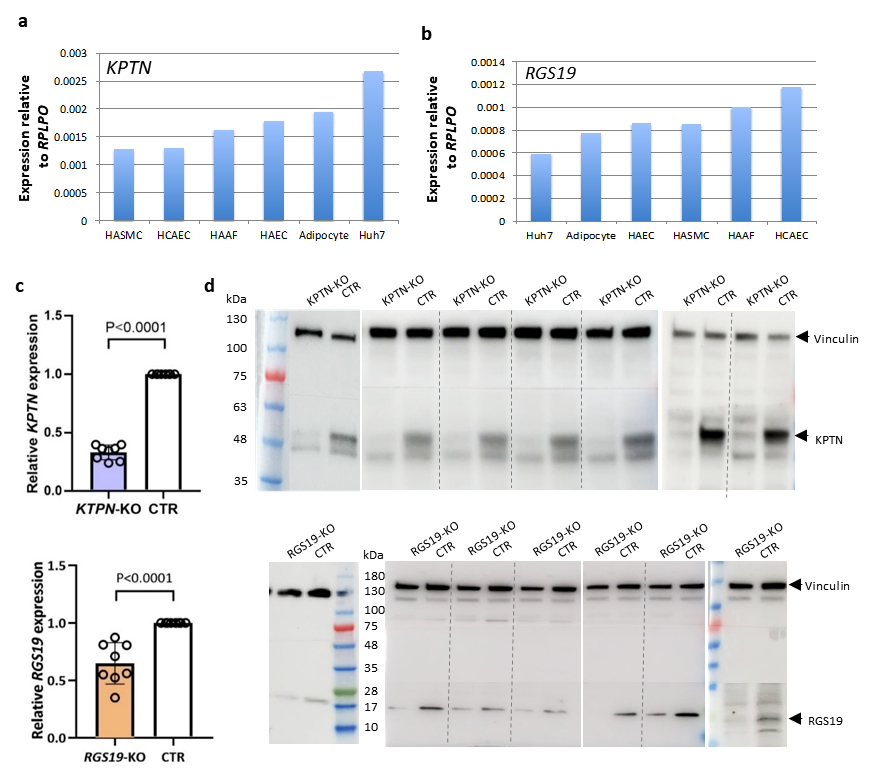


**Supplementary Fig. 11** *KPTN* (a) and *RGS19* (b) expressions in multiple primary cells and cell lines. (c) RNA levels of *KPTN* and *RGS19* were dramatically reduced in corresponding knockout lines (KO) in comparison to the control cell line (CTR), n=7. (d) The Western Blot image displays *KPTN* and *RGS19* reduction at protein level, n=7. Vinculin, 116kDa; *KPTN*, 48kDa; *RGS19*, 25kDa. HASMC, human aorta smooth muscle cell; HCAEC, human coronary artery endothelium cell; HAAF, human aorta artery fibroblast; HAEC, human aorta endothelium cell and huh7, a human hepatoma cell line.

**Supplementary Fig. 12** RNA-seq results of *KPTN*- and *RGS19*- knockout hepatocytes suggested potential mechanisms of CAD. (a, c) Top 10 ranking GO terms enriched by genes that are differentially expressed in *KPTN*- and *RGS19*- knockout (KO) hepatocytes as indicated (P≤ 9.55e-6 and P≤1.71e-5 respectively, n=3 per group). (b, d) Heatmap of genes related to potential pathways linking lipid phenotypes of the two genes. (Three replicates per group). The down-regulation of a gene indicated in blue, whereas up-regulation in red.
